# Stabilization of the [C_2_N_5_]^7–^ Anion in Recoverable High-Pressure Eu_4_Fe_0.864(6)_(C_2_N_5_)_2_ Pyronitridocarbonate

**DOI:** 10.1021/jacs.5c21756

**Published:** 2026-03-04

**Authors:** Fariia Iasmin Akbar, Nityasagar Jena, Christian Tobeck, Pascal L. Jurzick, Niko T. Flosbach, Valerio Cerantola, Elena Bykova, Lukas Brüning, Andrey Aslandukov, Dominik Spahr, Valentin Kovalev, Gaston Garbarino, Anna Pakhomova, Georgios Aprilis, Nico Giordano, Leonid Dubrovinsky, Mathias S. Wickleder, Uwe Ruschewitz, Igor A. Abrikosov, Maxim Bykov

**Affiliations:** † Institute of Inorganic and Analytical Chemistry, 9173 Goethe University Frankfurt, 60438 Frankfurt am Main, Germany; ‡ Department of Physics, Chemistry and Biology (IFM), 4566 Linköping University, SE-58183 Linköping, Sweden; § Institute of Inorganic and Materials Chemistry, 14309 University of Cologne, 50939 Cologne, Germany; ∥ Department of Earth and Environmental Sciences DISAT, 9305 University of Milano-Bicocca, I-20126 Milano, Italy; ⊥ Institute of Geosciences, 9173 Goethe University Frankfurt, 60438 Frankfurt, Germany; # 55553 European Synchrotron Radiation Facility, CS 40220, 38043 Grenoble Cedex 9, France; 7 28332 Deutsches Elektronen-Synchrotron DESY, Notkestrasse 85, 22607 Hamburg, Germany; 8 Bavarian Research Institute of Experimental Geochemistry and Geophysics (BGI), 26523 University of Bayreuth, 95440 Bayreuth, Germany

## Abstract

Synthesis at extreme conditions enables access to nitrogen-rich carbon–nitrogen anions that cannot be obtained at ambient conditions. Here, through a direct reaction between Eu­(N_3_)_2_ and EuC_2_ with Fe in a laser-heated diamond anvil cell (DAC) at 50(3) GPa, we synthesized the first inorganic hydrogen-free pyronitridocarbonate, Eu_4_Fe_*x*_(C_2_N_5_)_2_, *x* = 0.864(6), featuring novel highly charged [C_2_N_5_]^7–^ anions, along with the first stoichiometric oxygen-free rare-earth metal guanidinate Eu_5_(CN_3_)_3_. The crystal structures of both compounds were determined via synchrotron single-crystal X-ray diffraction (SCXRD) and fully corroborated by density functional theory (DFT) calculations. Eu_4_Fe_*x*_(C_2_N_5_)_2_ was found to be recoverable at pressures close to ambient. Keeping the sample at ambient conditions for 1 day leads to splitting of half of the [C_2_N_5_]^7–^ units in Eu_4_Fe_*x*_(C_2_N_5_)_2_ into the guanidinate [CN_3_]^5–^ and carbodiimide [CN_2_]^2–^ anions. The statistical analysis of the multigrain SCXRD data and DFT-based electronic structure analysis well defined the chemical nature of the bonding in [C_2_N_5_]^7–^ and [CN_3_]^5–^ anions. This study provides a clear synthetic pathway to a new class of inorganic nitridocarbonates.

## Graphical abstract

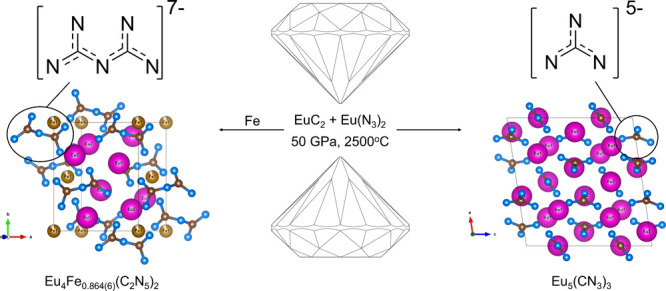

## Introduction

Synthesis and stabilization of compounds containing complex nitridocarbonate anions is an emerging topic in high-pressure chemistry, offering access to previously unknown bonding motifs and highly charged carbon–nitrogen species. Carbon–nitrogen anions occur in important classes of inorganic solids and include widely studied cyanides [CN]^−^ ^1^ and carbodiimides [CN_2_]^2–^,^2,3^ as well as less common acetonitriletriides [C_2_N]^3–^,^4,5^ dicyanamides [N­(CN)_2_]^−^,^6^ tricyanidomethanides [C­(CN)_3_]^−^,^7^ tetracyanoethylene radicals [C_2_(CN)_4_]^•–^,^8^ pentacyanoethanides [C_2_(CN)_5_]^−^,^9^ pentacyanopropenides [NCC­{C­(CN)_2_}_2_]^−^,^10,11^ guanidinates [CN_3_]^5–^,^12^ tricyanidomelaminates [C_6_N_9_]^3–^,^13^ melonates [C_6_N_7_(NCN)_3_]^3–^,^14^ cyanopolyynides [C_2*n*+1_N]^−^ (*n* = 0, 1, 2),^15^ cyano adduct anions of fullerenes [C_60_(CN)_*n*_]^−^ (*n* = 1, 3, 5),^16^ [C_60_(CN)_*n*_]^2–^ (*n* = 2, 4, 6),^17^ [C_70_(CN)_*n*_]^−^ (*n* = 1, 2, 3), and [C_70_(CN)_*n*_]^2–^ (*n* = 2, 4, 6).^18^ Ternary *M*–C–N compounds featuring nitrogen–nitrogen bonds are represented by the derivatives of tetrazole, e.g., 5-azidotetrazolate [CN_7_]^−^,^19^ 5,5′-azotetrazolate [N_2_(CN_4_)_2_]^2–^,^20,21^ and 4,5-bis­(tetrazol-5-*yl*)-2H-1,2,3-triazolate [C_2_N_3_(CN_4_)_2_]^3–^.^22^

Despite this structural diversity, most C–N anions remain nitrogen-poor. High-pressure synthesis is effective way to stabilize nitrogen-rich species, as was shown on the examples of various polynitrides.^23–30^ Recent high-pressure reactions in multicomponent C–N systems yielded compounds featuring guanidinate [CN_3_]^5–^,^31,32^ melaminate [C_3_N_6_]^6–^,^33,34^ as well as the poly-*N*-(1,3,5-triazin-2-*yl*)-guanidine anions in Bi_7_C_10_N_18_(N_3(1–*x*)_O_3*x*_).^35^ Syntheses at megabar pressure revealed the formation of polynitridocarbonates formed by anionic three-dimensional framework consisting of CN_4_ tetrahedra connected via di- or oligo-nitrogen linkers.^36^

Despite recent discoveries in ternary C–N-containing compounds, many potential forms of C–N anions remain unknown. A similar tendency to form a wide variety of species composed of two light elements has been identified in high-pressure carbonates, where pressure stabilizes pyrocarbonates containing [C_2_O_5_]^2–^ anions.^37–42^ We hypothesize that by analogy with a series of carbonate-pyrocarbonate species, the elusive pyronitridocarbonate anion [C_2_N_5_]^7–^ may also exist and be accessible in a high-pressure synthesis. The biggest challenge is to stabilize a relatively high odd-number charge in a rather small anion. Highly charged anions are typically found either in extended polyanionic frameworks (e.g., in Bi_7_C_10_N_18_(N_3(1–*x*)_O_3*x*_),^35^ LaCN_3_ and CeCN_5_ ^36^) or within large, isolated clusters ([P_3_N_9_]^12–^ in Li_12_P_3_N_9_, [P_4_N_10_]^10–^ in α-/β-Li_10_P_4_N_10_, and [B_3_P_3_N_13_]^15–^ in Li_47_B_3_P_14_N_42_).^43^ Small highly charged isolated anions are rare. Among examples are the ethanide anions [C_2_]^6–^ in Dy_3_C_2._ ^44,45^

In this work, we present the synthesis of the first inorganic hydrogen-free pyronitridocarbonate Eu_4_Fe_0.864(6)_(C_2_N_5_)_2_ featuring [C_2_N_5_]^7–^ anions—fully deprotonated derivatives of biguanide C_2_N_5_H_7_.^46^ Under similar conditions we also obtained the first stoichiometric oxygen-free rare-earth element nitridocarbonate, Eu_5_(CN_3_)_3_.

## Results and Discussion

We employed a new high-pressure synthetic strategy to access ternary and quaternary nitridocarbonates by laser-heating a mixture of europium azide Eu­(N_3_)_2_ and europium carbide EuC_2_ with a minor amount of iron in a DAC. Products of chemical reaction performed at 50(3) GPa and a temperature of 2800(200) K were identified by means of SCXRD at ESRF (ID15b, ID27) and DESY (P02.2) (see section Methods in the Supporting Information). The multigrain diffraction data analysis revealed the formation of several novel compounds in the laser-heated area. Full experimental details are provided in Tables S1–S6 and Figures S1–S4 of the Supporting Information. All supplemental results of the theoretical calculations are given in Tables S7–S10, Figures S5–S15, and Discussion S1. In the following sections, we describe the crystal structures and physical properties of two main products: Europium­(III) nitridocarbonate Eu_5_(CN_3_)_3_ and Europium­(III)–Iron­(II)-pyronitridocarbonate Eu_4_Fe_0.864(6)_(C_2_N_5_)_2_.

### Europium Nitridocarbonate Eu_5_(CN_3_)_3_

Europium guanidinate Eu_5_(CN_3_)_3_ crystallizes in the monoclinic space group *C*2/*c* (Figure 1, Table S2) and contains three crystallographically unique Eu atoms (Eu1 and Eu2 occupying the 8*f* Wyckoff site, and Eu3 on the 4*e* site). The structure features two symmetry-independent guanidinate anions [CN_3_]^5–^ with C–N distances varying in the range of ∼1.32–1.36 Å (Figure 1b), consistent with those reported for SbCN_3,_ ^31^ Ln_3_O_2_(CN_3_) (Ln = La, Eu, Gd, Tb, Ho, Yb),^32^ Sr_4_(Sr_6_N)_2_[In_4_]­[CN_3_]_4_, and (Sr_9_N_1.33(8)_)­(SrIn_3_)­[CN_3_],^12^ supporting the formal C–N bond order of 1.33. Europium atoms are coordinated by 9 or 10 nitrogen atoms, forming a distorted monocapped tetragonal antiprism (Eu1), a *cis*-bicapped cube (Eu2), and a tricapped trigonal prism (Eu3) (Figure S2a). Eu_5_(CN_3_)_3_ remains detectable via synchrotron XRD down to 16(2) GPa during decompression (Table S3).

**Figure 1:**
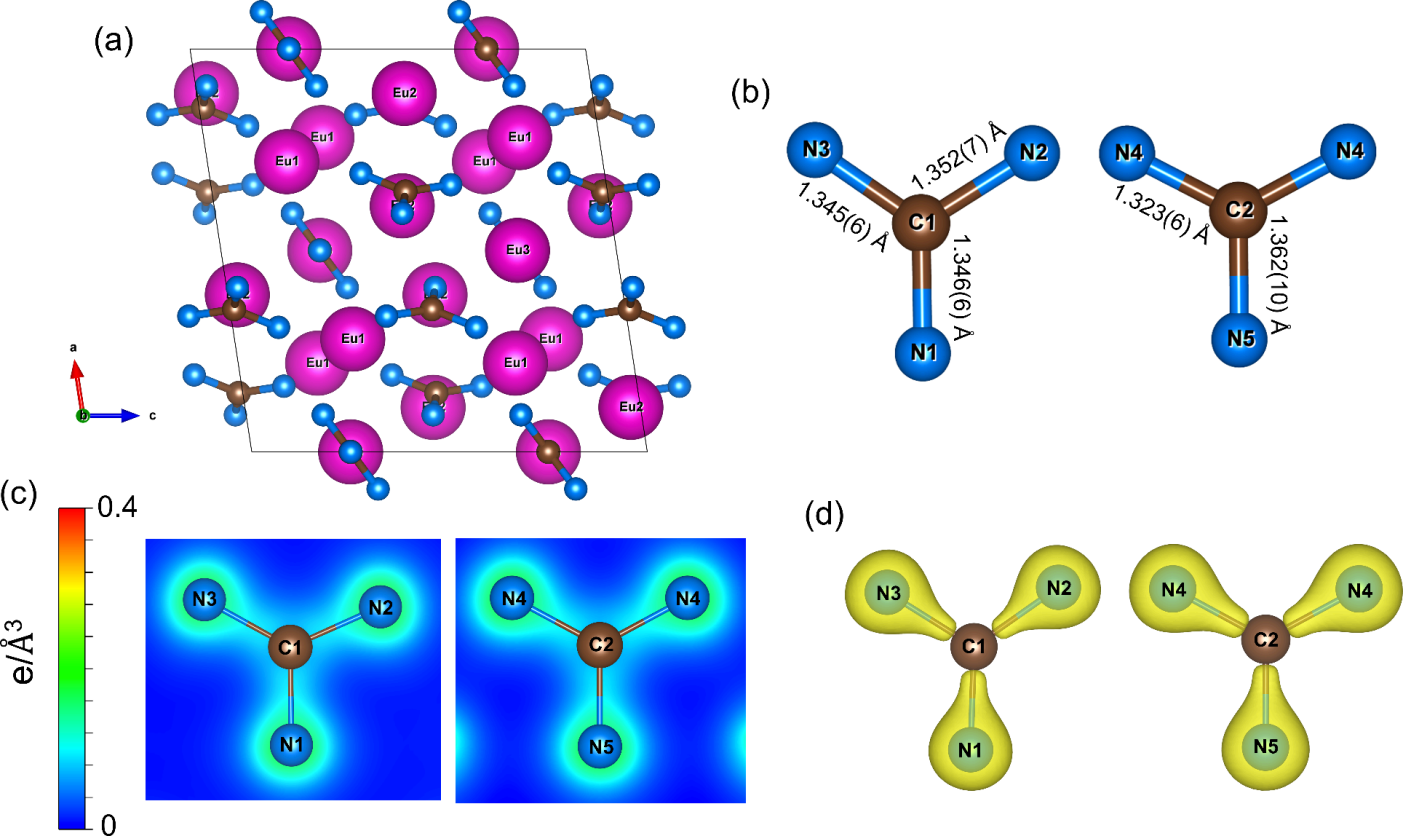
Crystal structure of europium guanidinate Eu_5_(CN_3_)_3_ at 50(3) GPa. All Eu atoms are purple, C atoms are brown, and N atoms are blue; gray thin lines outline the unit cell. (a) The unit cell in projection along the *b* axis. (b) A view of the [CN_3_]^5–^ units. (c) Charge density distribution in plains containing the [CN_3_]^5–^ unit atoms. (d) Charge density isosurfaces of the guanidinate anions (with an isosurface level of 0.3).

The experimental crystal structure of Eu_5_(CN_3_)_3_ was fully optimized at 50 GPa within the GGA+*U* framework using both the PBE+*U* and PBEsol+*U* functionals. The optimized lattice parameters at 50 GPa exhibit excellent agreement with experimental values obtained from SCXRD data with a deviation of less than 2% (Table S8).

A BM3 EoS was used to describe volume–pressure dependence obtained from DFT calculations in the range of 0–60 GPa, yielding equilibrium unit cell volumes of 823 Å^3^ and 785 Å^3^ using PBE+*U* and PBEsol+*U* functionals, respectively, with an estimated bulk modulus *K*_0_ in the range of 130–155 GPa (Figure 2a). Lattice parameters of Eu_5_(CN_3_)_3_ increase monotonically upon pressure release over the entire pressure range without any sign of lattice distortions (Figure S5a). Phonon dispersion relations calculated using the harmonic approximation (Figure S6) indicate dynamical instabilities in Eu_5_(CN_3_)_3_ both at the synthesis pressure and at 1 bar, attributed to C–N bond asymmetry in the [CN_3_]^5–^ units. This instability is lifted by using finite-temperature phonon dispersion calculations at *T* = 300 K via the sTDEP method (Figure 3a, b).

**Figure 2:**
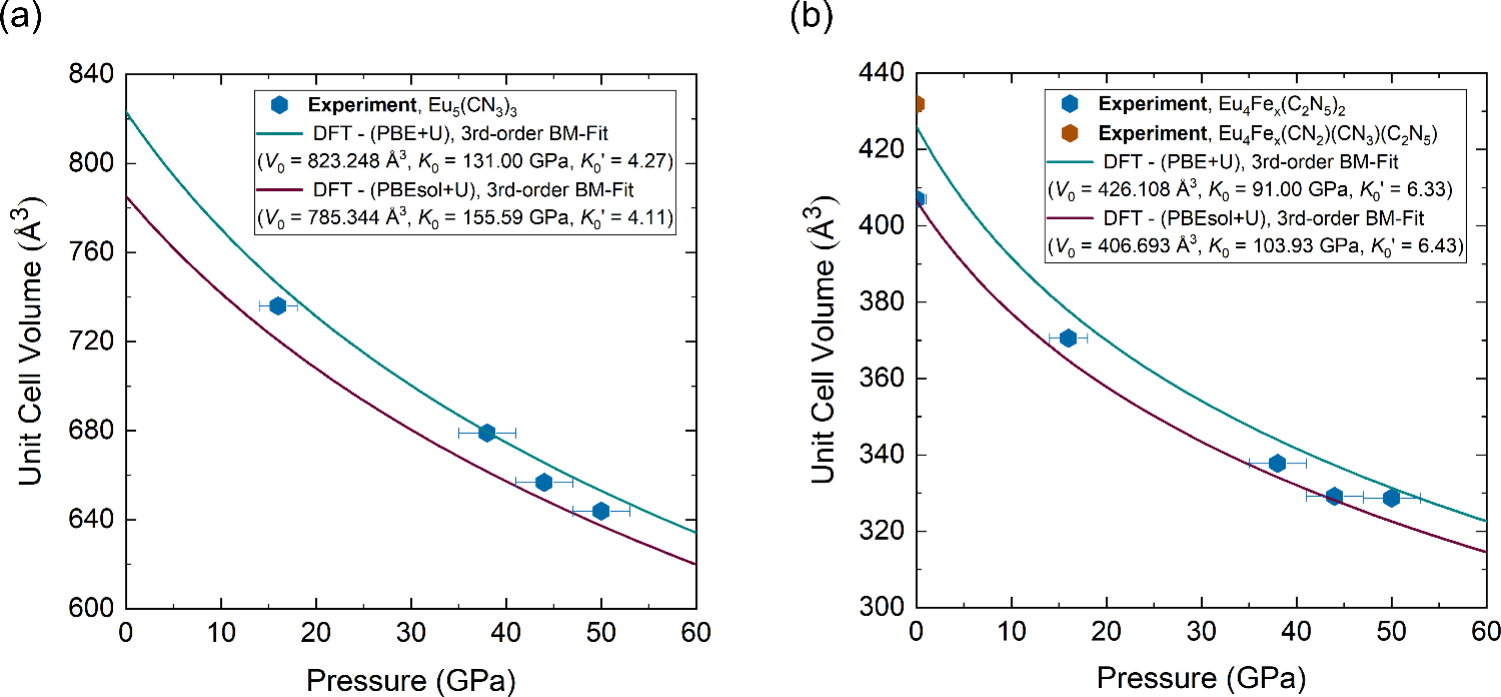
(a) The pressure dependence of the unit cell volume of Eu_5_(CN_3_)_3_ over a pressure range of 0–60 GPa, calculated using both PBE+*U* and PBEsol+*U* functionals within the DFT+*U* framework, compared with the experimental data. Solid lines correspond to the third-order Birch–Murnaghan Equation of State (BM3 EoS) based on DFT calculations. (b) The pressure dependence of the unit cell volume of Eu_4_Fe­(C_2_N_5_)_2._ The experimental data at ambient conditions for the Eu_4_Fe­(CN_2_)­(CN_3_)­(C_2_N_5_) is marked by different color (brown).

**Figure 3:**
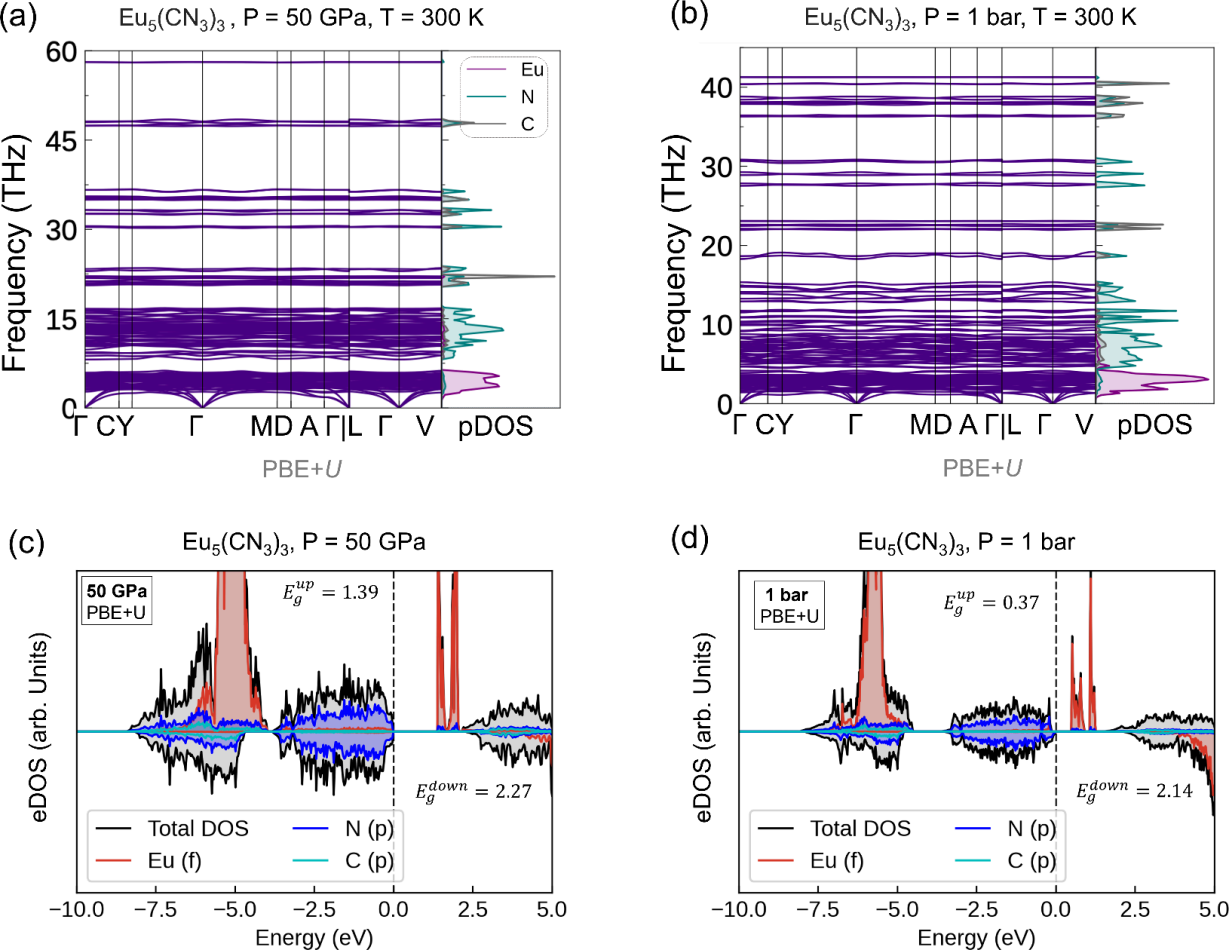
(a)–(b) Finite temperature phonon dispersion relations and the phonon density of states (pDOS) for Eu_5_(CN_3_)_3_ at 50 GPa, and 1 bar, respectively, calculated using the sTDEP methods at *T* = 300 K. (c)–(d) Spin-resolved electronic density of states (eDOS) for Eu_5_(CN_3_)_3_ at 50 GPa and 1 bar, respectively, calculated using PBE+*U* functional.

Within the PBE+*U* calculations, at 50 GPa, Eu_5_(CN_3_)_3_ is an insulator with an energy gap between the occupied N 2*p*-states and unoccupied highly localized Eu 4*f* states of 1.39 eV for the spin-up channel and 2.27 eV for the spin-down channel (Figure 3c). Comparable bandgap values were obtained using the PBEsol+*U* functional (Table S8, Figures S7a and S8b). Eu_5_(CN_3_)_3_ remains insulating over the entire pressure range from 50 GPa to 1 bar (Figure 3c, d).

The occupied Eu 4*f* states lie at ∼6 eV below the Fermi energy, and nitrogen 2*p* orbitals dominate the valence band edge. The bandgap of Eu_5_(CN_3_)_3_ decreases monotonically upon decompression with an increasing discrepancy between PBE+*U* and PBEsol+*U* bandgap energy values (Figure S7a). Each europium atom in Eu_5_(CN_3_)_3_ carries a calculated magnetic moment of 6 μ_*B*_, with unoccupied localized 4*f* states near the conduction band edge comprising one *f*-electron from each europium atom.

Although the finite-temperature (*T* = 300 K) phonon dispersion relation calculated for Eu_5_(CN_3_)_3_ at ambient pressure shows the lattice dynamic stability of the compound (Figure 3b), it was not observed in our SCXRD experiments below a pressure of 16 GPa (Figure 2a).

### Europium–Iron Pyronitridocarbonate Eu_4_Fe_*x*_(C_2_N_5_)_2_

Europium–iron pyronitridocarbonate, Eu_4_Fe_*x*_(C_2_N_5_)_2_, *x* = 0.864(6) (space group *P*2_1_/*c*) (Table S4), contains an unprecedented pyronitridocarbonate [C_2_N_5_]^7–^ anion (Figures 4 and S3), representing the fully deprotonated form of biguanide C_2_N_5_H_7_.^46^ Europium, iron, nitrogen, and carbon atoms in Eu_4_Fe_*x*_(C_2_N_5_)_2_ occupy the following Wyckoff sites: 4*e* (Eu1, Eu2, N1–N5, C1, and C2) and 2*a* (Fe1) (Table S4). Two symmetry-independent Eu atoms, Eu1 and Eu2, are coordinated by 10 and 11 nitrogen atoms, respectively, forming a distorted bicapped tetragonal antiprism and a fully capped trigonal prism. Fe1 atoms feature octahedral coordination (Figure S2b).

**Figure 4:**
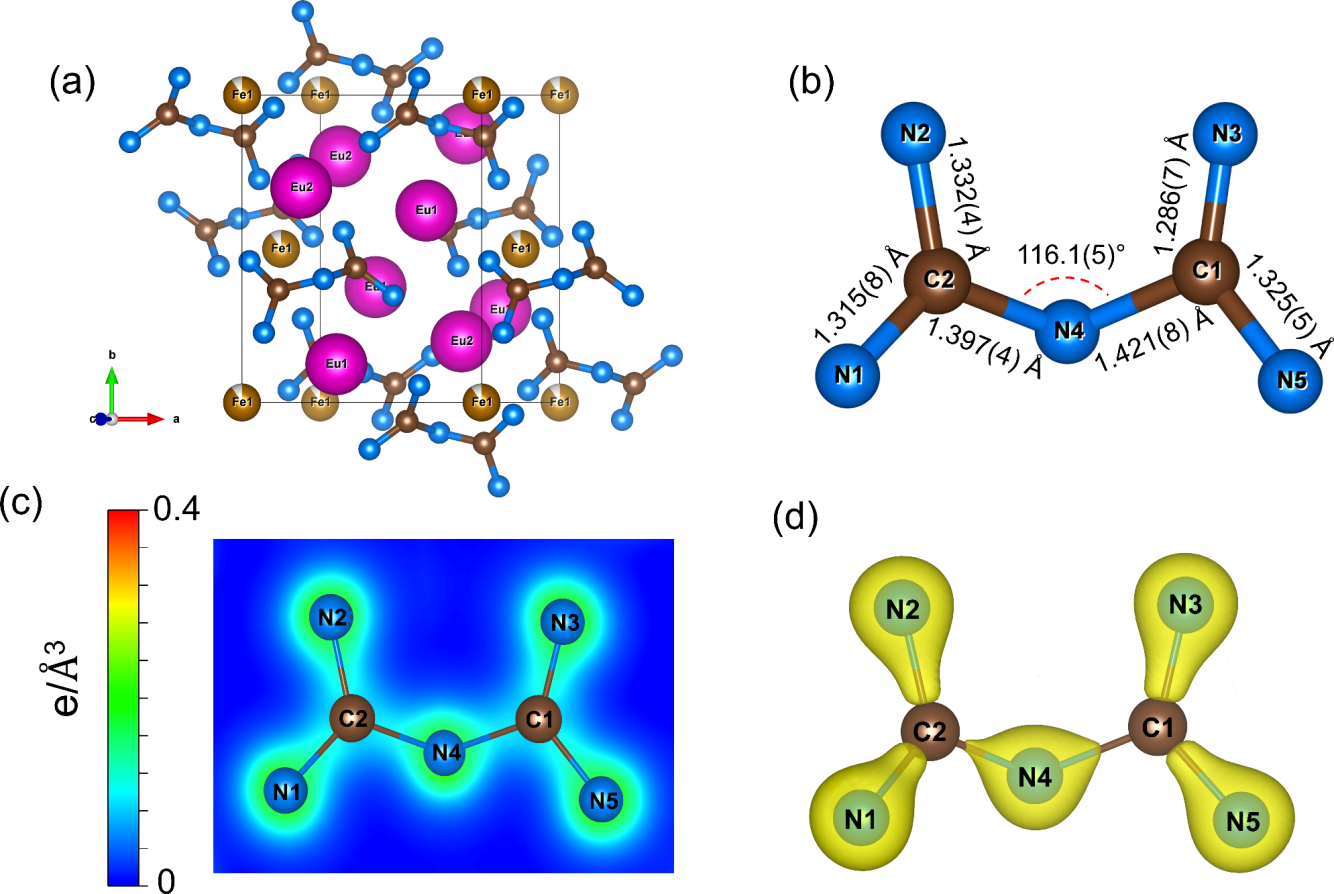
Crystal structure of europium pyronitridocarbonate Eu_4_Fe_*x*_(C_2_N_5_)_2_ at 50(3) GPa. Eu atoms are purple, Fe atoms are yellow, C atoms are brown, and N atoms are blue; gray thin lines outline the unit cell. (a) A general view of the crystal structure of Eu_4_Fe_*x*_(C_2_N_5_)_2_. (b) A pyronitridocarbonate anion geometry. (c) Charge density distribution in plains containing the [C_2_N_5_]^7–^ unit atoms. (d) Charge density isosurface of the pyronitridocarbonate anion (with an isosurface level of 0.3).

The C–N distances in the [C_2_N_5_]^7–^ units of Eu_4_Fe_*x*_(C_2_N_5_)_2_, determined at 50(3) GPa, are equal to ∼1.40–1.42 Å for bridging nitrogen atoms and ∼1.29–1.33 Å for the terminal nitrogen atoms (Figure 4b). Both distance ranges indicate an intermediate state between a single C–N bond (∼1.47 Å^47^) and a bond of order 1.5, as observed in pyridine (*d*_C–N_ ∼ 1.34 Å ^48,49^ ). Moreover, bond orders derived from the resonance forms of the biguanide molecule C_2_N_5_H_7_ ^46^ are consistent with the C–N distances in the [C_2_N_5_]^7–^ anions: 1.25 for the bridging atom and 1.375 for the terminal ones. The charge density distribution clearly shows the difference between the bridging and terminal C–N bonds in [C_2_N_5_]^7–^ (Figure 4c, d), specifically, the peripheral bonds show a more pronounced electron density (Figure 4c, d). The geometry of the observed nonplanar [C_2_N_5_]^7–^ anions (Figures 4b and S3) can be compared to those in pyrocarbonates, possessing a wide variety of anion geometries.^37–42^

The partial occupancy *x* = 0.864(6) of Fe in Eu_4_Fe_*x*_(C_2_N_5_)_2_, along with the interatomic C–N distances, was determined through the statistical analysis of 26 individual structure refinements (as described in the Methods section). The Mössbauer spectrum of Eu_4_Fe_*x*_(C_2_N_5_)_2_ at 50(3) GPa represents a singlet component with a center shift of 0.26 mm/s (Figure S4), in agreement with a low-spin state of Fe^2+^ in an octahedral coordination.^50,51^ The low signal intensity is attributed to the fact that the sample was prepared with naturally occurring iron rather than ^57^Fe-enriched iron, which is typically used in high-pressure experiments exploiting Mössbauer spectroscopy as an analytical technique. The deviation from 100% Fe site occupancy requires the presence of a fraction of Fe^III^ for the charge balance (e.g., Eu_3_Fe^II^_0.592_Fe^III^_0.272_(C_2_N_5_)_2_). The Fe^III^ component, regardless of its spin state, may lie below the detection limit of Mössbauer spectroscopy due to the low signal-to-noise ratio and overlap with the signal from the beamline optics and is therefore not clearly observable in the spectrum. Nonstoichiometry in iron-containing compounds, where iron is present predominantly in the +II oxidation state with a smaller proportion in the +III state, is well-known, as exemplified by wüstite Fe_1–*x*_O.^52,53^

Decompression of the DAC containing the sample demonstrated the recoverability of Eu_4_Fe_*x*_(C_2_N_5_)_2_ down to ambient pressure (Tables S5 and S6). After ca. 1 day under ambient conditions, half of the [C_2_N_5_]^7–^ anions split into guanidinate [CN_3_]^5–^ and carbodiimide [CN_2_]^2–^ anions (Figures 5b–d and S3), resulting in an abrupt increase of the unit cell volume and symmetry lowering (Table S6) in the compound, whose chemical formula can be written as Eu_4_Fe_*x*_(CN_2_)­(CN_3_)­(C_2_N_5_).

**Figure 5:**
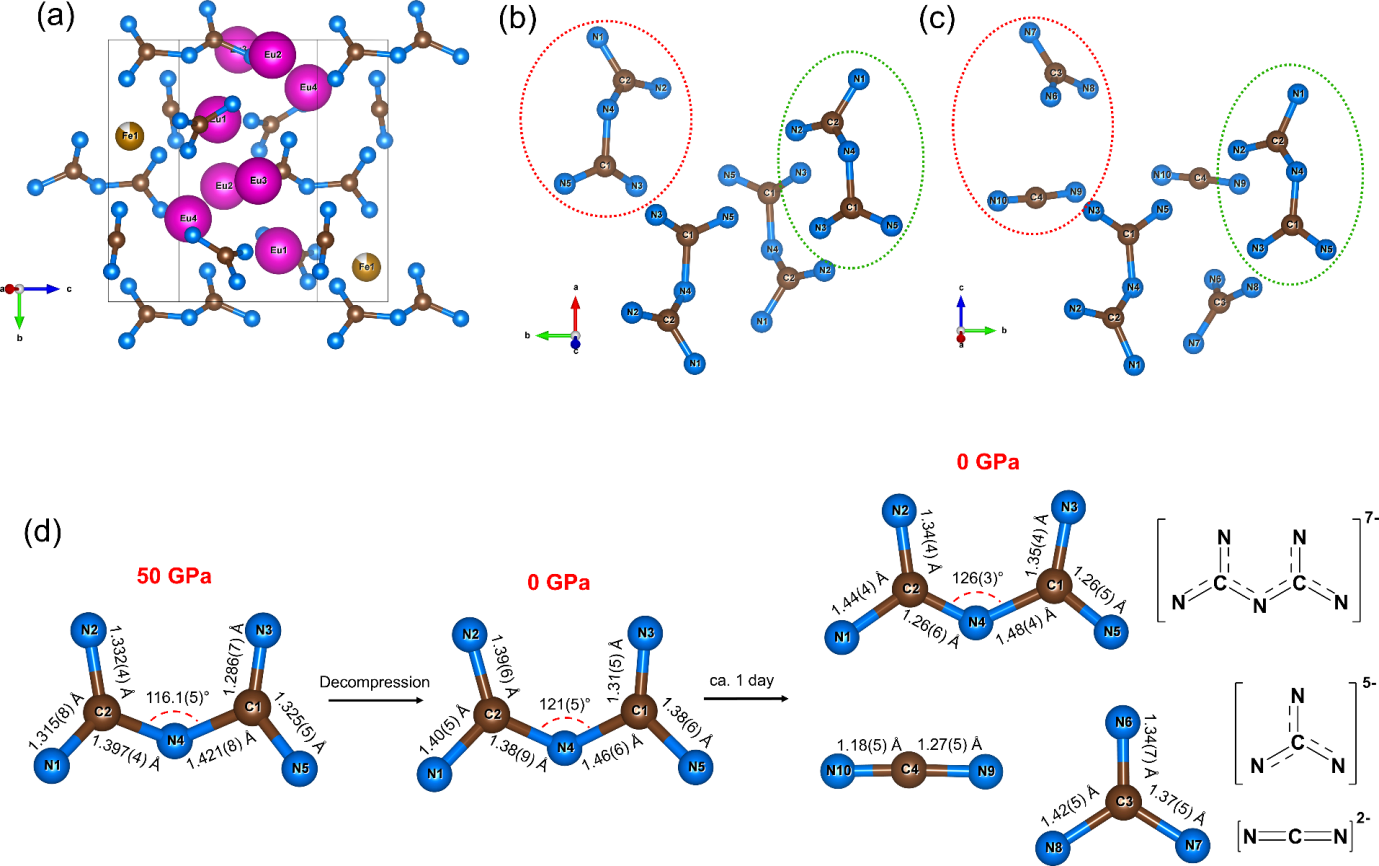
Crystal structure of the europium pyronitridocarbonate after chemical transformation to Eu_4_Fe_*x*_(CN_2_)­(CN_3_)­(C_2_N_5_) under ambient conditions. Eu atoms are purple, Fe atoms are yellow, C atoms are brown, and N atoms are blue; gray thin lines outline the unit cell. (a) A general view of the crystal structure of Eu_4_Fe_*x*_(CN_2_)­(CN_3_)­(C_2_N_5_). (b) A view of the [C_2_N_5_]^7–^ units before chemical transformation. (c) A view of the C–N anions after chemical transformation at ambient conditions. (d) Schematic representation of the [C_2_N_5_]^7–^ anion splitting into guanidinate [CN_3_]^5–^ and carbodiimide [CN_2_]^2–^ anions.

The DFT+*U* optimized crystal structure of Eu_4_Fe­(C_2_N_5_)_2_ (space group *P*2_1_/*c*) at 50 GPa shows excellent agreement with the experimental crystal structure obtained from SCXRD (Tables S4 and S9). In the DFT calculations, we used an idealized chemical composition with *x* = 1 for both Eu_4_Fe_*x*_(C_2_N_5_)_2_ and Eu_4_Fe_*x*_(CN_2_)­(CN_3_)­(C_2_N_5_). At ambient pressure, the calculated unit cell volume of Eu_4_Fe­(CN_2_)­(CN_3_)­(C_2_N_5_) differs from experiment by ∼5% with PBE+*U*, whereas PBEsol+*U* significantly improves the agreement with an error of less than 1.3% (Figure 2b, Table S9).

A BM3 fit to the pressure–volume data of Eu_4_Fe­(C_2_N_5_)_2_ yields an equilibrium unit cell volume of 426 Å^3^ and 406 Å^3^ using PBE+*U* and PBEsol+*U*, respectively (Figure 2b, with an estimated bulk modulus in the range of 90–100 GPa). The lattice parameters calculated within PBE+*U* closely match the experiment at 50 GPa, whereas PBEsol+*U* provides better agreement at ambient pressure. At 50 GPa, Eu_4_Fe­(C_2_N_5_)_2_ is dynamically stable as shown in Figure 6a. The experimental unit cell volumes at the intermediate pressure range 45–15 GPa fall between the PBE+*U* and PBEsol+*U* predictions (Figure 2b). Additionally, the lattice parameters (*a*, *b*, and *c*) of Eu_4_Fe­(C_2_N_5_)_2_ show a smooth, monotonous decrease over the pressure range 10–50 GPa (Figure S5b). Below 10 GPa, lattice distortions emerge, indicating potential phase transformations from Eu_4_Fe­(C_2_N_5_)_2_ to Eu_4_Fe­(CN_2_)­(CN_3_)­(C_2_N_5_). This interpretation is further supported by phonon dispersion calculations for Eu_4_Fe­(C_2_N_5_)_2_ at ambient pressure, which reveal imaginary phonon modes associated with Fe–N lattice vibrations, indicative of dynamic instability in Eu_4_Fe­(C_2_N_5_)_2_ (Figure S9). Conversely, the Eu_4_Fe­(CN_2_)­(CN_3_)­(C_2_N_5_) phase remains dynamically stable at ambient pressure (Figure 6b), supporting experimental observations.

**Figure 6:**
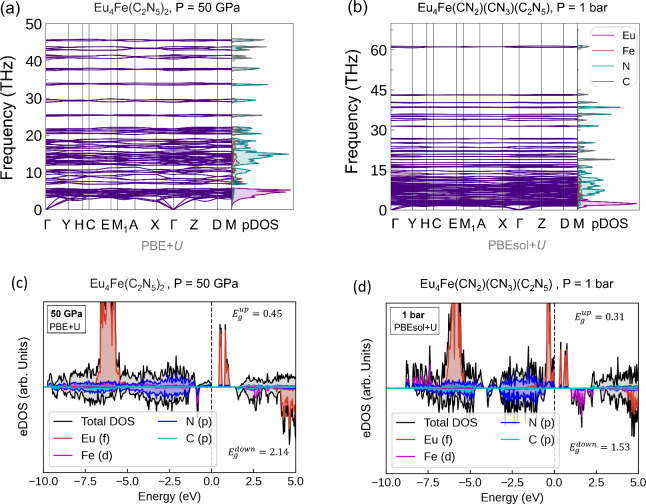
(a)–(b) Phonon dispersion relations and pDOS for Eu_4_Fe­(C_2_N_5_)_2_ at 50 GPa and Eu_4_Fe­(CN_2_)­(CN_3_)­(C_2_N_5_) at 1 bar using harmonic approximations. (c)–(d) Spin-resolved eDOS of Eu_4_Fe­(C_2_N_5_)_2_ at 50 GPa and Eu_4_Fe­(CN_2_)­(CN_3_)­(C_2_N_5_) at 1 bar calculated with the PBE+*U* and PBEsol+*U* functionals, respectively.

Figure 6c shows the spin-polarized total and partial eDOS of Eu_4_Fe­(C_2_N_5_)_2_ at 50 GPa, using the PBE+*U* functional. The phase exhibits an insulating behavior in both majority (spin-up) and minority (spin-down) spin channels, with energy gaps of 0.45 and 2.14 eV, respectively. The occupied Eu 4*f* states lie ∼ −6 eV below the Fermi level, while the unoccupied 4*f* states reside within the conduction band. Strong Fe 3*d* – N 2*p* hybridization occurs below the Fermi level for the spin-down channel, whereas in the spin-up channel the occupied Fe 3*d* states are distributed over a wider energy range of about 8 eV. The fundamental energy gap between the occupied (valence band) states and the unoccupied (conduction band) states is 0.45 eV, with unoccupied Eu 4*f* states exhibiting two distinct peaks, subject to a large exchange splitting induced by the localized moments of Eu 4*f* states with an energy width of 0.7 eV. A more detailed analysis of the eDOS and magnetic ordering for Eu_4_Fe­(C_2_N_5_)_2_ is provided in Supplementary Discussion S1.

Figure 6d shows the spin-polarized eDOS for Eu_4_Fe­(CN_2_)­(CN_3_)­(C_2_N_5_) at 1 bar, using PBEsol+*U*. It exhibits a band gap of 0.31 eV for the spin-up channel and 1.53 eV for the spin-down channel. In Eu_4_Fe­(CN_2_)­(CN_3_)­(C_2_N_5_), the occupied Eu 4*f* states appear at two distinct energy positions below the Fermi level: the majority of these states are positioned at −6 eV, while a smaller fraction appears just below the Fermi energy. A detailed analysis of the Wyckoff site resolved eDOS suggests that the Eu 4*f* states at −6 eV primarily originate from three europium sublattices (Eu1­(2*a*), Eu2­(2*a*), and Eu4­(2*a*)), whereas the occupied states just below the Fermi level arise from a single Eu3­(2*a*) sublattice (Figure S10d). This suggests a partial reduction of one Eu^3+^ ion to Eu^2+^ and oxidation of iron from +II to +III in Eu_4_Fe­(CN_2_)­(CN_3_)­(C_2_N_5_).

## Conclusions

High-pressure synthesis at 50(3) GPa enabled the stabilization of fully deprotonated guanidinate [CN_3_]^5–^ and pyronitridocarbonate [C_2_N_5_]^7–^ anions in Eu_5_(CN_3_)_3_ and Eu_4_Fe_*x*_(C_2_N_5_)_2_ (*x* = 0.864(6)), respectively. The [C_2_N_5_]^7–^ anion represents a key new member of the *sp* ^2^-hybridized C–N polyanion family, positioned between simple nitridocarbonates [CN_3_]^5–^ and melaminates [C_3_N_6_]^6–^ (Figure 7). These results highlight the importance of precise pressure tuning and strategic selection of countercations for the design of highly charged nitridocarbonate species. While [CN_3_]^5–^ can be stabilized by the group 5 and group 15 cations (e.g., Sb as shown experimentally for SbCN_3_,^31^ or V, Nb, Ta as predicted for (V,Nb,Ta)­CN_3_ ^54^), stabilization of anions with the formal ionic charge of 7– is much more challenging and requires either a single element in a mixed-valence state, or a selected combination of two cations. In the case of [C_2_N_5_]^7–^, such stabilization was achieved by a combination of the Eu and Fe ions. This finding opens new directions for nitridocarbonate chemistry under extreme conditions.

**Figure 7:**
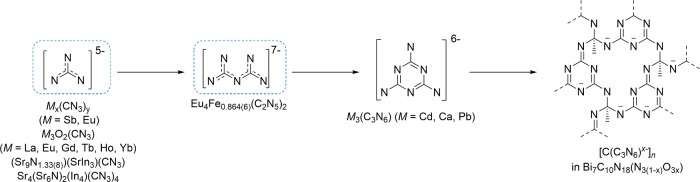
Schematic representation of the family of nitridocarbonates featuring progressive condensation of CN_3_ building blocks: [CN_3_]^5–^,^12,31,32^ [C_2_N_5_]^7–^, [C_3_N_6_]^6–^,^33,34^ and [C_4_N_6_^*x*–^]_*n*_.^35^

Traditional high-pressure routes to *M*–C–N phases often rely on metal–nitrogen reactions with carbon provided by the diamond anvils or involve C–N precursors such as cyanuric triazide (C_3_N_12_).^31,32,35^ Our approach employs precursors that already contain oxidized metals with fixed *M*:C and *M*:N ratios, offering more opportunities for stoichiometry control.

Eu_4_Fe_*x*_(C_2_N_5_)_2_ significantly expands the chemistry of ternary and quaternary *M*–C–N compounds. Hitherto, only very few quaternary *RE*–Fe–C–N compounds had been explored, namely *RE*_2_Fe_17_CN_*x*_ (*RE* = Y, Sm, Gd, Tb, Dy, and Er)^55^ and NdFe­(CN)_6_.^56^ The former compounds are interstitial carbonitrides, while the latter is a cyanide. Among ternary rare earth-containing nitridocarbonate compounds, only carbodiimides *RE*_2_(CN_2_)_3_ (*RE* = Sc,^57^ Sm,^58^ Yb,^59^ Lu^58,60^ ), carbodiimide nitrides Ce_3_(CN_2_)_3_N,^61^ cyanamides EuCN_2_,^62^ and dicyanamides *RE*[N­(CN)_2_]_3_ (*RE* = La,^6^ Ce,^6^ Pr,^6^ Nd,^6^ Sm,^6^ Eu,^6^ and Gd^63^) are known to date.

Finally, decompression of Eu_4_Fe_*x*_(C_2_N_5_)_2_ resulted in an interesting example of *in crystallo* chemistry: a single-crystal-to-single-crystal transformation in which 50% of the [C_2_N_5_]^7–^ anions split into [CN_2_]^2–^ and [CN_3_]^5–^ anions while preserving the main structural motifs.

## Supplementary Material



## Notes

Equations used in the statistical analysis of the C–N anions; crystallographic information; Synchrotron Mössbauer Source spectrum; supplementary DFT calculation results; supplementary discussion of theoretical computations (DOCX)
