# Supplementary material for: Stabilization of the [C2N5]7– Anion in Recoverable High-Pressure Eu4Fe0.864(6)(C2N5)2 Pyronitridocarbonate
Source: J Am Chem Soc. 2026 Mar 4;148(11):11915–24. doi: 10.1021/jacs.5c21756 (PMC13022862; doi:10.1021/jacs.5c21756)
Supplement: Supplementary file 1 [file ja5c21756_si_001.docx]

# **Supporting Information**

**Stabilization of C_2_N_5_^7-^ anion in recoverable high-pressure Eu_4_Fe_0.864(6)_(C_2_N_5_)_2_ pyronitridocarbonate**

Fariia Iasmin Akbar^1^, Nityasagar Jena^2^, Christian Tobeck^3^, Pascal L. Jurzick^1^, Niko T. Flosbach^3^, Valerio Cerantola^4^, Elena Bykova^5^, Lukas Brüning^1^, Andrey Aslandukov^1^, Dominik Spahr^5^, Valentin Kovalev^5^, Gaston Garbarino^6^, Anna Pakhomova^6^, Georgios Aprilis^6^, Nico Giordano^7^, Leonid Dubrovinsky^8^, Mathias S. Wickleder^3^, Uwe Ruschewitz^3^, Igor A. Abrikosov^2^, Maxim Bykov^1^

^1^ Institute of Inorganic and Analytical Chemistry, Goethe University Frankfurt, 60438, Frankfurt am Main, Germany

^2^ Department of Physics, Chemistry and Biology (IFM), Linköping University, SE-58183, Linköping, Sweden

^3^ Institute of Inorganic and Materials Chemistry, University of Cologne, 50939 Cologne, Germany

^4^ Department of Earth and Environmental Sciences DISAT, University of Milano-Bicocca, I-20126 Milano, Italy

^5^ Institute of Geosciences, Goethe University Frankfurt, 60438 Frankfurt, Germany

^6^ European Synchrotron Radiation Facility, CS 40220, 38043 Grenoble Cedex 9, France

^7^ Deutsches Elektronen-Synchrotron DESY, Notkestrasse 85, 22607 Hamburg, Germany

^8^ Bavarian Research Institute of Experimental Geochemistry and Geophysics (BGI), University of Bayreuth, 95440 Bayreuth, Germany

***Corresponding authors:** Fariia Iasmin Akbar akbar@chemie.uni-frankfurt.de; Maxim Bykov [maxim.bykov@chemie.uni-frankfurt.de](mailto:maxim.bykov@chemie.uni-frankfurt.de); Nityasagar Jena nityasagar.jena@liu.se.

***Keywords:*** high-pressure, diamond anvil cell, rare-earth nitridocarbonates, rare-earth elements, europium.

**Methods**

**Sample preparation**

In our experiments, we used the BX90-type diamond anvil cell with a large X-ray aperture ^1^. We employed Boehler−Almax-type diamonds with culets diameter of 250 μm. Rhenium gasket with an initial thickness of 200 μm was indented to ~28 μm and a hole of ~100 μm laser-drilled in the center of the indentation. The mixture of europium azide Eu(N_3_)_2_ (synthesized as a result of the chemical reaction between europium (II) triflate and tetrabutylammonium azide in acetonitrile ^2^) and europium dicarbide EuC_2_ was loaded into the pressure chamber under an inert atmosphere of Ar in the glovebox. During rasping of the europium pieces for the synthesis of EuC_2_, small amounts of an iron impurity were introduced into this sample, although according to the powder X-ray diffraction data, the sample was phase-pure, *i.e*., neither EuO nor elemental Fe were detectable. In a typical synthesis^3^, 0.154 g Eu (1.013 mmol, freshly rasped in a glovebox prior to reaction) and 0.0255 g graphite (2.123 mmol, heated at 800 °C under vacuum for 24 hours prior to reaction) were pressed into a pellet and sealed in a purified Ta ampule under inert conditions (800 mbar He). This Ta ampule was flame-sealed in a quartz ampule (Ar atmosphere) and heated at 1200 °C for 24 hours (cooling to room temperature within 12 hours). The Eu(N_3_)_2_/EuC_2_ mixture was compressed to 50(3) GPa and laser-heated up to 2800(200) K using in house double-sided YAG laser (λ = 1064 nm) heating systems of BGI (University of Bayreuth, Bayreuth, Germany) ^4,5^. The temperature during laser heating was determined by the blackbody radiation fit. The initial pressure in the sample chamber, as well as pressure upon decompression of the cell, was determined by the Raman signal from the diamond anvils ^6^ and additionally monitored by the diffraction of Re gasket edge using the equation of state (EoS) of Re ^7^.

**X-ray diffraction**

The X-ray diffraction (XRD) measurements were conducted at the ID15b ^8^ (λ = 0.4099 Å, beam size ~1 × 1 μm^2^) and ID27 ^9^ (λ = 0.3738 Å, beam size ~2 × 2 μm^2^) beamlines of ESRF, Grenoble, France, and P02.2 (λ = 0.2900 Å, beam size ~ 2 × 2 μm^2^) beamline of Petra III, DESY, Hamburg, Germany. At ID15b and ID27, the XRD data was collected on an Eiger2X CdTe 9M hybrid photon-counting pixel detector, and at P02.2, the diffraction patterns were collected on a PerkinElmer 1621 XRD flat-panel detector.

In order to determine the position of the polycrystalline sample on which the SCXRD acquisition was obtained, an X-ray diffraction mapping of the pressure chamber was performed. The sample areas producing the highest number of strong single-crystal reflections, corresponding to the phases of interest, were selected for data collection in step-scans of 0.5° from −36° to +36°. The CrysAlis^Pro^ software package ^10^ was used for the analysis of the SCXRD data (peak hunting, indexing, data integration, frame scaling, and absorption correction). To calibrate an instrument model in the CrysAlis^Pro^ software, i.e. the sample-to-detector distance, detector’s origin, offsets of the goniometer angles, and rotation of both the X-ray beam and detector around the instrument axis, we used a single crystal of orthoenstatite [(Mg_1.93_,Fe_0.06_)(Si_1.93_,Al_0.06_)O_6_, *Pbca* space group, *a* = 8.8117(2) Å, *b* = 5.18320(10) Å, and *c* = 18.2391(3) Å]. The DAFi program was used to sort reflections into groups belonging to the individual single-crystal domains ^11^. Using the OLEX2 software package ^12^, the structures were solved with the ShelXT structure solution program ^13^ using intrinsic phasing and refined with the ShelXL ^14^ refinement package using least-squares minimization. Crystal structure visualization was made with the VESTA software ^15^. The equations of state were obtained by fitting the pressure-volume dependence data using the EoSFit7-GUI ^16^. The analysis of polyhedra was performed using Polynator software ^17^.

In order to improve both precision and accuracy in determining the geometry of the structural units, 26–28 single-crystal domains with R_int_ < 5% and for which the individual structure refinements converged with R_1_ < 8% were identified and used in the statistical analysis of bond distances and angles (see Table S1).

The full crystallographic datasets for all compounds have been deposited in the Cambridge Structural Database (CSD) at the Cambridge Crystallographic Data Centre (CCDC) ^18^, and their details with corresponding deposition numbers are provided in Tables S2–S6 and Figures S1–S3.

**Mössbauer spectroscopy**

Synchrotron Mössbauer Source (SMS) spectra were recorded at the Nuclear Resonance Beamline ID14 of the ESRF ^19^ using the (111) nuclear reflection of a ^57^FeBO_3_ single crystal mounted on a Wissel velocity transducer driven with a sinusoidal waveform ^20^. The X-ray beam was focused down to 6 × 8 μm^2^ (V × H) using Kirkpatrick-Baez mirrors. The linewidth of the SMS and the absolute position of the center shift relative to α-iron was controlled several times during the course of the experiment using a K_2_Mg^57^Fe(CN)_6_ reference single line absorber. The velocity scale was calibrated using 25 μm thick natural α-iron foil. The collection time of the spectrum presented in supplementary Figure S4 was ~5 hours. The spectrum was fitted using a transmission integral taking into account the exact shape of the SMS instrumental function. The fitting procedure was done in SYNCmoss software package ^21^. The analysis results of the SMS spectrum are shown in Figure S4.

**Theoretical calculations details**

The electronic structure properties of the synthesized compounds were determined through the first-principles calculations in the framework of DFT as implemented in the Vienna Ab initio Simulation Package (VASP version 6.4.3) ^22^ using the Projector-Augmented-Wave (PAW) method and a planewave basis set for the electronic wavefunctions ^23,24^. The generalized gradient approximation (GGA) functionals, specifically Perdew–Burke–Ernzerhof (PBE) ^25^ and its variant revised for solids PBEsol ^26,27^, were used to calculate the exchange-correlation energies with a kinetic energy cutoff of 600 eV and free energy convergence threshold of 10^-6^ eV. The geometry optimization was using the conjugate gradient relaxation scheme with variable-cell relaxations at a target pressure until the forces on each ion were less than 10^-3^ eV/Å and the residual stress below 0.1 GPa. A Gaussian smearing scheme with an energy width of 0.05 eV was used in the geometry optimization. The Brillouin-zone integrations were using a 6×6×3 k-point mesh for Eu_5_(CN_3_)_3_ and a 7×5×7 k-point mesh for Eu_4_Fe(C_2_N_5_)_2_ and Eu_4_Fe(CN_2_)(CN_3_)(C_2_N_5_) according to the Monkhorst-Pack scheme ^28^. The electronic density of states (eDOS) was calculated using the tetrahedron method with Blöchl corrections without any smearing with k-point resolution of 0.02 Å^-1^ ^29^. The PAW potentials and the valence electronic configurations for each atomic species are shown in Table S7. DFT+*U* formalism was used to treat the localized 4*f*-electrons of Eu, and 3*d*-electrons of Fe atoms as formulated by Dudarev *et al.* to account for the on-site Coulomb repulsions in the studied compounds ^30^ using both PBE+*U* and PBEsol+*U* exchange-correlation functionals.

The DFT+*U* formalism, which accounts for the static electronic correlation effects, provides an effective framework for modeling the electronic structure properties of strongly correlated systems, including the europium-based compounds ^31^. While more advanced approaches, such as DFT combined with dynamical mean-field theory (DFT+DMFT) or variational wavefunction-based methods, offer a more accurate treatment of electronic correlation effects in rare-earth compounds, these high-level methods fall beyond the scope of the present study and will be explored in future theoretical investigations. In Dudarev’s formalism, only the difference between *U* (Coulomb repulsion term) and *J* (Hubbard exchange term) is significant, thus, *U* – *J* is simply denoted as *U* or *U_eff_* (Table S7)*.* Unless otherwise stated, the effective Hubbard *U* term (*U_eff_* = *U* – *J*), as defined by Dudarev’s approach, was set to 7 eV for Eu and 5 eV for Fe in our DFT+*U* calculations. Similar values of Hubbard *U* parameters have been used to describe the on-site Coulomb interactions among Eu *f*-orbitals and Fe *d*-orbitals in previous studies ^31–33^. For all studied compounds, spin-polarized calculations were used to account for possible local magnetic moments in a ferromagnetic spin configuration for the magnetic atoms using the collinear method (Tables S8-S10, Discussion S1). Harmonic phonon dispersion relations were obtained using the finite-displacement method, with symmetry-imposed supercell displacement geometries generated by the Phonopy code ^34,35^. The interatomic forces in these displacement supercells were computed using VASP in DFT+*U* formalism. For Eu_5_(CN_3_)_3_ a 3×3×2 supercell (612 atoms) and 2×2×2 k-mesh, for Eu_4_Fe(C_2_N_5_)_2_ a 2×2×2 supercell (304 atoms) and 2×2×2 k-mesh, and for Eu_4_Fe(CN_2_)(CN_3_)(C_2_N_5_) a 2×2×2 supercell (304 atoms) and 2×2×2 k-mesh were used for the phonon calculations with atomic displacement amplitudes of 0.01 Å in the finite-displacement method. The finite temperature phonon calculations at *T* = 300 K were used for the Eu_5_(CN_3_)_3_ case to account for its anharmonic vibrational behavior of [CN_3_]^5-^ units using the stochastically initialized Temperature Dependent Effective Potential method (sTDEP) ^36–38^.

**Experimental results**

**Table S1.** Equations used in the statistical analysis of the geometry of C–N anions in Eu_5_(CN_3_)_3_ and Eu_4_Fe_x_(C_2_N_5_)_2_, as well as the Fe content in Eu_4_Fe_x_(C_2_N_5_)_2_.

| Weighted average | $\bar{a}=\frac{w_{1}a_{1}+w_{2}a_{2}+\ldots+w_{n}a_{n}}{w_{1}+w_{2}+\ldots+w_{n}}$ | *a_i_* – a value from experiment |
| --- | --- | --- |
| Weight (reciprocal of variance) | $w_{i}=\frac{1}{\sigma_{i}^{2}}$ | $\sigma_{i}$ – standard uncertainty (experimental error) |
| Weighted standard deviation (quantifies how spread out individual measurements are) | $SD\left[ \bar{a} \right]=\sqrt{\frac{n}{n-1}\frac{\sum_{i}^{n} w_{i}\left( a_{i}-\bar{a} \right)^{2}}{\sum_{i}^{n} w_{i}}}$ |  |
| Weighted standard error (quantifies how well the sample mean estimates the population mean) | $SE\left[ \bar{a} \right]=\frac{SD\left[ \bar{a} \right]}{\sqrt{n}}$ |  |

**Table S2.** Crystallographic information for a selected domain of mC68 Eu_5_(CN_3_)_3_ at 50(3) GPa and the corresponding DFT-relaxed structure data. The assigned Pearson symbol refers to the arrangement of the fully occupied atoms in a unit cell. The crystallographic dataset for Eu_5_(CN_3_)_3_ at 50(3) GPa was deposited to the CCDC under the deposition number 2495723.

| Chemical formula | | Eu_5_(CN_3_)_3_ (experiment) | Eu_5_(CN_3_)_3_ (PBE+*U*) |
| --- | --- | --- | --- |
| Pressure (GPa) | | 50(3) | 50 |
| *Crystal data* | | | |
| Mr | | 921.92 |  |
| ρ (g/cm^3^) | | 9.510 |  |
| Radiation type | | X-ray, λ = 0.2900 Å |  |
| Space group | | *C*2/*c*, #15 | |
| a (Å) | | 10.318(14) | 10.3064 |
| b (Å) | | 6.3045(7) | 6.3790 |
| c (Å) | | 10.0150(16) | 10.0370 |
| β (°) | | 98.72(4) | 98.75 |
| V (Å^3^) | | 643.9(9) | 652.20 |
| Z | | 4 | |
| CN of Eu1 | | 9 | |
| CN of Eu2 | | 10 | |
| CN of Eu3 | | 9 | |
| Eu1–N distances in first coordination sphere (Å) | | 2.265(15) – 2.80(4) | 2.2734 – 2.6790 |
| Eu2–N distances in first coordination sphere (Å) | | 2.276(14) – 3.075(13) | 2.3034 – 3.06154 |
| Eu3–N distances in first coordination sphere (Å) | | 2.260(13) – 2.471(17) | 2.2996 – 2.49344 |
| Atom / Wyck. site / Fractional atomic coordinates  (x; y; z) | Eu1/8*f* | 0.2201(2) 0.24095(10) 0.20089(8) | 0.22040 0.24110 0.19980 |
|  | Eu2/8*f* | 0.1105(2) 0.13773(11) 0.47576(8) | 0.11130 0.13700 0.47540 |
|  | Eu3/4*e* | 0 0.60348(15) 0.25 | 0 0.60410 0.25 |
|  | N1/8*f* | 0.408(4) 0.251(2) 0.0271(15) | 0.39610 0.24950 0.02870 |
|  | N2/8*f* | 0.191(3) 0.4472(15) 0.3841(11) | 0.18400 0.44640 0.38450 |
|  | N3/8*f* | 0.337(4) 0.0605(19) 0.3934(15) | 0.33310 0.05970 0.39430 |
|  | N4/8*f* | 0.408(3) 0.4325(17) 0.3050(12) | 0.40700 0.43390 0.30320 |
|  | N5/4*e* | 0 0.245(2) 0.25 | 0 0.24360 0.25 |
|  | C1/8*f* | 0.354(4) 0.0525(18) 0.0106(13) | 0.35310 0.05010 0.01120 |
|  | C2/4*e* | 0 0.030(3) 0.25 | 0 0.03250 0.25 |
| *Data collection* | | | |
| No. of measured, independent and observed [I > 2σ(I)] reflections | | 1403/715/571 |  |
| R_int_ | | 3.40% |  |
| *Refinement* | | | |
| R_1_ | | 5.29% |  |
| wR_2_ | | 14.15% |  |
| GOF | | 1.061 |  |
| No. of reflections/No. of parameters | | 715/48 |  |

**Table S3.** Crystallographic information for selected domains of *mC*68 Eu_5_(CN_3_)_3_ at decompression steps: 44(3) GPa, 38(3) GPa, and 16(2) GPa. The crystallographic datasets for Eu_5_(CN_3_)_3_ at 44(3) GPa, 38(3) GPa, and 16(2) GPa were deposited to the CCDC under the deposition numbers 2495725, 2495719, and 2495720, respectively.

| Chemical formula | | Eu_5_(CN_3_)_3_ | | |
| --- | --- | --- | --- | --- |
| Pressure (GPa) | | 44(3) | 38(3) | 16(2) |
| *Crystal data* | | | | |
| Mr | | 921.92 | | |
| ρ (g/cm^3^) | | 9.325 | 9.019 | 8.322 |
| Radiation type | | X-ray, λ = 0.2900 Å | | X-ray, λ = 0.3738 Å |
| Space group | | *C*2/*c*, #15 | | |
| a (Å) | | 10.182(12) | 10.340(16) | 10.99(4) |
| b (Å) | | 6.4509(9) | 6.5194(11) | 6.472(6) |
| c (Å) | | 10.156(2) | 10.1763(16) | 10.426(9) |
| β (°) | | 100.11(4) | 98.23(5) | 97.1(2) |
| V (Å^3^) | | 656.7(8) | 678.9(11) | 736(3) |
| Z | | 4 | | |
| CN of Eu1 | | 9 | | |
| CN of Eu2 | | 10 | | 9 |
| CN of Eu3 | | 9 | | |
| Eu1–N distances in first coordination sphere (Å) | | 2.30(6) – 2.71(10) | 2.26(4) – 2.94(8) | 2.16(13) – 2.84(10) |
| Eu2–N distances in first coordination sphere (Å) | | 2.26(5) – 3.19(5) | 2.20(8) – 3.17(3) | 2.36(7) – 2.99(12) |
| Eu3–N distances in first coordination sphere (Å) | | 2.34(6) – 2.51(5) | 2.32(6) – 2.49(4) | 2.27(12) – 2.65(13) |
| Atom / Wyck. site / Fractional atomic coordinates  (x; y; z) | Eu1/8*f* | 0.2198(6) 0.2410(3) 0.2012(2) | 0.2198(5) 0.2392(3) 0.19974(15) | 0.2156(6) 0.2373(5) 0.2034(4) |
|  | Eu2/8*f* | 0.1076(6) 0.1406(3) 0.4753(2) | 0.1104(5) 0.1418(3) 0.47744(15) | 0.1174(6) 0.1405(5) 0.4843(4) |
|  | Eu3/4*e* | 0 0.6040(4) 0.25 | 0 0.6009(4) 0.25 | 0 0.6004(6) 0.25 |
|  | N1/8*f* | 0.395(11) 0.255(6) 0.029(4) | 0.428(8) 0.232(5) 0.031(3) | 0.406(10) 0.252(8) 0.042(6) |
|  | N2/8*f* | 0.190(10) 0.455(5) 0.385(4) | 0.180(7) 0.451(4) 0.384(2) | 0.161(10) 0.441(9) 0.371(6) |
|  | N3/8*f* | 0.344(9) 0.057(5) 0.401(4) | 0.333(10) 0.050(5) 0.392(3) | 0.332(7) 0.054(6) 0.394(4) |
|  | N4/8*f* | 0.410(9) 0.430(5) 0.311(4) | 0.401(7) 0.432(4) 0.305(2) | 0.380(17) 0.450(14) 0.277(11) |
|  | N5/4*e* | 0 0.242(9) 0.25 | 0 0.245(8) 0.25 | 0 0.250(18) 0.25 |
|  | C1/8*f* | 0.363(8) 0.042(4) 0.012(3) | 0.334(8) 0.044(4) 0.006(3) | 0.148(7) 0.445(6) 0.006(4) |
|  | C2/4*e* | 0 0.034(9) 0.25 | 0 0.013(9) 0.25 | 0 0.053(8) 0.25 |
| *Data collection* | | | | |
| No. of measured, independent and observed [I > 2σ(I)] reflections | | 866/385/299 | 935/400/298 | 361/331/222 |
| R_int_ | | 5.37% | 4.20% | 4.51% |
| *Refinement* | | | | |
| R_1_ | | 8.94% | 6.28% | 11.80% |
| wR_2_ | | 25.36% | 17.74% | 33.68% |
| GOF | | 1.099 | 1.104 | 1.389 |
| No. of reflections/No. of parameters | | 385/48 | 400/48 | 331/48 |

**Table S4.** Crystallographic information for a selected domain of mP38 Eu_4_Fe_x_(C_2_N_5_)_2_ at 50(3) GPa and the corresponding DFT-relaxed structure data. The assigned Pearson symbol refers to the arrangement of the fully occupied atoms in a unit cell. The crystallographic dataset for Eu_4_Fe_x_(C_2_N_5_)_2_ at 50(3) GPa was deposited to the CCDC under the deposition number 2495724.

| Chemical formula | | Eu_4_Fe_x_(C_2_N_5_)_2_ (experiment) | Eu_4_Fe(C_2_N_5_)_2_ (PBE+*U*) |
| --- | --- | --- | --- |
| Pressure (GPa) | | 50(3) | 50 |
| *Crystal data* | | | |
| Mr | | 846.80 |  |
| ρ (g/cm^3^) | | 8.555 |  |
| Radiation type | | X-ray, λ = 0.4099 Å |  |
| Space group | | *P*2_1_/*c*, #14 | |
| a (Å) | | 6.675(16) | 6.6718 |
| b (Å) | | 8.1521(14) | 8.2282 |
| c (Å) | | 6.361(3) | 6.3262 |
| β (°) | | 108.26(12) | 107.81 |
| V (Å^3^) | | 328.7(8) | 330.65 |
| Z | | 2 | |
| CN of Eu1 | | 10 | |
| CN of Eu2 | | 11 | |
| CN of Fe1 | | 6 | |
| Eu1–N distances in first coordination sphere (Å) | | 2.33(3) – 2.546(11) | 2.3031 – 2.5221 |
| Eu2–N distances in first coordination sphere (Å) | | 2.344(16) – 3.04(3) | 2.3169 – 3.0495 |
| Fe1–N distances in first coordination sphere (Å) | | 1.926(15) – 1.957(12) | 1.9844 – 2.0238 |
| Atom / Wyck. site / Fractional atomic coordinates  (x; y; z) | Eu1/4*e* | 0.3388(2) 0.37547(6) 0.33037(8) | 0.33799 0.37486 0.33268 |
|  | Eu2/4*e* | 0.7846(2) 0.30101(6) 0.09710(8) | 0.78409 0.30221 0.09539 |
|  | Fe1/2*a* | 0 0 0 | 0 0 0 |
|  | N1/4*e* | 0.553(4) 0.1511(9) 0.2376(13) | 0.54353 0.15111 0.23332 |
|  | N2/4*e* | 0.297(4) 0.5559(10) 0.6207(13) | 0.30792 0.56017 0.62412 |
|  | N3/4*e* | 0.037(4) 0.5391(10) 0.2159(14) | 0.04627 0.53950 0.20899 |
|  | N4/4*e* | 0.323(4) 0.0987(10) 0.4399(14) | 0.32305 0.09451 0.44343 |
|  | N5/4*e* | 0.092(4) 0.2279(10) 0.0002(15) | 0.08470 0.23337 0.00526 |
|  | C1/4*e* | 0.104(5) 0.1321(10) 0.4042(17) | 0.10804 0.13056 0.40487 |
|  | C2/4*e* | 0.397(5) 0.0627(12) 0.2639(17) | 0.39213 0.05928 0.26256 |
| Atom site occupancy | Fe1 | 0.909(16) | 1 |
| *Data collection* | | | |
| No. of measured, independent and observed [I > 2σ(I)] reflections | | 841/460/417 |  |
| R_int_ | | 1.76% |  |
| *Refinement* | | | |
| R_1_ | | 3.73% |  |
| wR_2_ | | 9.89% |  |
| GOF | | 1.048 |  |
| No. of reflections/No. of parameters | | 460/54 |  |

**Table S5.** Crystallographic information for selected domains of mP38 Eu_4_Fe_x_(C_2_N_5_)_2_ at decompression steps: 44(3) GPa, 38(3) GPa, and 16(2) GPa. The assigned Pearson symbol refers to the arrangement of the fully occupied atoms in a unit cell. The crystallographic datasets for Eu_4_Fe_x_(C_2_N_5_)_2_ at 44(3) GPa, 38(3) GPa, and 16(2) GPa were deposited to the CCDC under the deposition numbers 2495721, 2495722, and 2495718, respectively.

| Chemical formula | | Eu_4_Fe_x_(C_2_N_5_)_2_ | Eu_4_Fe_x_(C_2_N_5_)_2_ | Eu_4_Fe_x_(C_2_N_5_)_2_ |
| --- | --- | --- | --- | --- |
| Pressure (GPa) | | 44(3) | 38(3) | 16(2) |
| *Crystal data* | | | | |
| Mr | | 842.06 | 839.26 | 845.41 |
| ρ (g/cm^3^) | | 8.498 | 8.253 | 7.577 |
| Radiation type | | X-ray, λ = 0.2900 Å | | X-ray, λ = 0.3738 Å |
| Space group | | *P*2_1_/*c*, #14 | | |
| a (Å) | | 6.735(15) | 6.787(10) | 6.811(9) |
| b (Å) | | 8.225(3) | 8.283(3) | 8.5601(12) |
| c (Å) | | 6.213(8) | 6.257(8) | 6.6451(19) |
| β (°) | | 107.0(2) | 106.23(16) | 106.98(7) |
| V (Å^3^) | | 329.1(9) | 337.7(7) | 370.5(5) |
| Z | | 2 | | |
| CN of Eu1 | | 10 | 10 | |
| CN of Eu2 | | 11 | 10 | |
| CN of Fe1 | | 6 | 6 | |
| Eu1–N distances in first coordination sphere (Å) | | 2.333(17) – 2.560(18) | 2.35(5) – 2.59(4) | 2.33(5) – 2.74(3) |
| Eu2–N distances in first coordination sphere (Å) | | 2.32(3) – 3.08(3) | 2.36(4) – 2.83(4) | 2.34(4) – 3.09(4) |
| Fe1–N distances in first coordination sphere (Å) | | 1.92(3) – 1.97(3) | 1.93(6) – 2.04(6) | 1.99(3) – 2.16(5) |
| Atom / Wyck. site / Fractional atomic coordinates  (x; y; z) | Eu1/4*e* | 0.3391(2) 0.37424(7) 0.33077(19) | 0.3376(6) 0.37431(18) 0.3309(5) | 0.3314(4) 0.37799(9) 0.32479(17) |
|  | Eu2/4*e* | 0.7840(3) 0.30350(6) 0.0886(2) | 0.7840(6) 0.30291(19) 0.0865(5) | 0.7779(4) 0.29819(10) 0.10057(17) |
|  | Fe1/2*a* | 0 0 0 | 0 0 0 | 0 0 0 |
|  | N1/4*e* | 0.541(4) 0.1510(10) 0.232(3) | 0.545(9) 0.151(3) 0.228(8) | 0.556(6) 0.1519(15) 0.238(3) |
|  | N2/4*e* | 0.295(4) 0.5583(12) 0.622(3) | 0.304(9) 0.557(3) 0.606(8) | 0.324(7) 0.5492(18) 0.629(3) |
|  | N3/4*e* | 0.050(3) 0.5444(10) 0.217(3) | 0.051(9) 0.545(3) 0.218(8) | 0.040(8) 0.5358(16) 0.218(3) |
|  | N4/4*e* | 0.324(4) 0.0949(11) 0.445(4) | 0.303(9) 0.096(3) 0.439(9) | 0.299(7) 0.1005(15) 0.440(3) |
|  | N5/4*e* | 0.084(4) 0.2747(11) 0.503(3) | 0.088(7) 0.274(2) 0.500(6) | 0.071(7) 0.261(2) 0.488(3) |
|  | C1/4*e* | 0.100(4) 0.1359(12) 0.403(3) | 0.115(10) 0.129(3) 0.395(8) | 0.130(7) 0.1273(19) 0.397(3) |
|  | C2/4*e* | 0.393(4) 0.0628(13) 0.263(4) | 0.387(11) 0.061(4) 0.268(10) | 0.389(8) 0.066(2) 0.261(3) |
| Atom site occupancy | Fe1 | 0.817(16) | 0.74(4) | 0.88(2) |
| *Data collection* | | | | |
| No. of measured, independent and observed [I > 2σ(I)] reflections | | 1054/652/498 | 830/423/343 | 1001/662/460 |
| R_int_ | | 2.90% | 3.68% | 2.90% |
| *Refinement* | | | | |
| R_1_ | | 4.24% | 8.88% | 6.48% |
| wR_2_ | | 10.65% | 26.13% | 16.81% |
| GOF | | 1.036 | 1.093 | 0.965 |
| No. of reflections/No. of parameters | | 652/54 | 423/39 | 662/54 |

**Table S6.** Crystallographic information for *mP*38 Eu_4_Fe_x_(C_2_N_5_)_2_ at the last decompression pressure step (0 GPa) and at ambient conditions ~24 h after chemical transformation to *mP*38 Eu_4_Fe_x_(CN_2_)(CN_3_)(C_2_N_5_), with the corresponding DFT-relaxed structure data. The assigned Pearson symbol refers to the arrangement of the fully occupied atoms in a unit cell. The crystallographic datasets for selected domains of Eu_4_Fe_x_(C_2_N_5_)_2_ and Eu_4_Fe_x_(CN_2_)(CN_3_)(C_2_N_5_) at 0 GPa were deposited to the CCDC under the deposition numbers 2495726 and 2495727, respectively.

| Chemical formula | | Eu_4_Fe_x_(C_2_N_5_)_2_  (experiment) | | Eu_4_Fe(C_2_N_5_)_2_  (PBEsol+*U*) | Eu_4_Fe_x_(CN_2_) (CN_3_)(C_2_N_5_)  (experiment) | | Eu_4_Fe(CN_2_) (CN_3_)(C_2_N_5_)  (PBEsol+*U*) |
| --- | --- | --- | --- | --- | --- | --- | --- |
| Pressure (GPa) | | 0 | | 0 | 0 | | 0 |
| *Crystal data* | | | | | | | |
| Mr | | 847.64 | |  | 844.01 | |  |
| ρ (g/cm^3^) | | 6.917 | |  | 6.490 | |  |
| Radiation type | | X-ray,  λ = 0.3738 Å | |  | X-ray,  λ = 0.3738 Å | |  |
| Space group | | *P*2_1_/*c*, #14 | | | *P*2_1_, #4 | | |
| a (Å) | | 7.043(5) | | 7.0854 | 6.880(5) | | 6.8291 |
| b (Å) | | 8.8048(15) | | 8.7953 | 9.0990(7) | | 9.2170 |
| c (Å) | | 6.893(14) | | 6.8501 | 7.2599(19) | | 7.2201 |
| β (°) | | 107.81(15) | | 107.38 | 108.14(5) | | 108.18 |
| V (Å^3^) | | 407.0(9) | | 407.41 | 431.9(3) | | 431.76 |
| Z | | 2 | | | | | |
| CN of Eu1 | | 10 | |  | 8 | |  |
| CN of Eu2 | | 9 | |  | 7 | |  |
| CN of Eu3 | |  | |  | 10 | |  |
| CN of Eu4 | |  | |  | 8 | |  |
| CN of Fe1 | | 6 | |  | 5+1 | |  |
| Eu1–N distances in first coordination sphere (Å) | | 2.43(3) – 2.97(6) | | 2.4817 – 2.8903 | 2.41(6) – 2.75(5) | | 2.4825 – 2.7486 |
| Eu2–N distances in first coordination sphere (Å) | | 2.40(5) – 2.947(19) | | 2.4782 – 2.8753 | 2.28(3) – 2.74(5) | | 2.3212 – 2.7051 |
| Eu3–N distances in first coordination sphere (Å) | |  | |  | 2.48(3) – 3.00(3) | | 2.4856 – 2.9187 |
| Eu4–N distances in first coordination sphere (Å) | |  | |  | 2.39(4) – 2.75(4) | | 2.3792 – 2.7379 |
| Fe1–N distances in first coordination sphere (Å) | | 1.90(7) – 2.22(3) | | 1.9871 – 2.1637 | 2.008(17) – 2.26(3), 2.60(3) | | 1.9645 – 2.1671, 2.6989 |
| Atom / Wyck. site / Fractional atomic coordinates  (x; y; z) | | Eu1/ 4*e* | 0.3280(2) 0.38192(11) 0.3175(5) | 0.33722 0.38224 0.32193 | Eu1/ 2*a* | 0.5614(4) 0.26601(11) 0.37496(19) | 0.55978 0.26448 0.37914 |
|  |  | Eu2/ 4*e* | 0.7777(3) 0.29039(13) 0.1182(5) | 0.77926 0.29358 0.11352 | Eu2/ 2*a* | 0.1439(3) 0.52019(10) 0.26266(19) | 0.14367 0.51816 0.26409 |
|  |  |  |  |  | Eu3/ 2*a* | 0.0627(4) 0.00000(10) 0.3033(2) | 0.06425 0 0.30410 |
|  |  |  |  |  | Eu4/ 2*a* | 0.3546(4) 0.14458(11) 0.73809(19) | 0.35481 0.13442 0.73589 |
|  |  | Fe1/ 2*a* | 0 0 0 | 0 0 0 | Fe1/ 2*a* | 0.7413(15) 0.3292(5) 0.0154(8) | 0.73648 0.32327 0.01958 |
|  |  | N1/ 4*e* | 0.558(4) 0.1473(17) 0.255(9) | 0.54416 0.14794 0.24597 | N1/ 2*a* | 0.499(6) 0.0251(17) 0.518(3) | 0.49932 0.02026 0.51306 |
|  |  | N2/ 4*e* | 0.312(4) 0.545(2) 0.613(7) | 0.30464 0.54098 0.62378 | N2/ 2*a* | 0.405(5) 0.8433(16) 0.273(3) | 0.39535 0.83724 0.27711 |
|  |  | N3/4*e* | 0.022(4) 0.5322(17) 0.235(9) | 0.03019 0.53681 0.22467 | N3/ 2*a* | 0.472(7) 0.4013(19) 0.063(4) | 0.48357 0.40415 0.06137 |
|  |  | N4/ 4*e* | 0.318(5) 0.117(2) 0.414(9) | 0.31157 0.11289 0.42228 | N4/ 2*a* | 0.641(6) 0.0208(18) 0.266(3) | 0.63885 0.02352 0.25839 |
|  |  | N5/ 4*e* | 0.056(5) 0.253(2) 0.478(9) | 0.06031 0.26105 0.47285 | N5/ 2*a* | 0.291(7) 0.6100(17) 0.010(3) | 0.28374 0.61145 0.01057 |
|  |  |  |  |  | N6/ 2*a* | 0.193(7) 0.2707(19) 0.411(4) | 0.20334 0.27136 0.41035 |
|  |  |  |  |  | N7/ 2*a* | 0.039(7) 0.7066(19) 0.418(3) | 0.03402 0.70266 0.42710 |
|  |  |  |  |  | N8/ 2*a* | 0.859(8) 0.384(2) 0.335(4) | 0.86501 0.38088 0.32462 |
|  |  |  |  |  | N9/ 2*a* | 0.027(7) 0.3459(19) 0.993(4) | 0.02636 0.34557 0.99287 |
|  |  |  |  |  | N10/ 2*a* | 0.205(9) 0.110(2) 0.016(4) | 0.20167 0.11021 0.01179 |
|  |  | C1/ 4*e* | 0.104(5) 0.1272(18) 0.384(9) | 0.10424 0.13372 0.38694 | C1/ 2*a* | 0.629(7) 0.007(2) 0.059(4) | 0.62205 0.00617 0.06216 |
|  |  | C2/ 4*e* | 0.388(4) 0.066(2) 0.260(9) | 0.38659 0.07004 0.25985 | C2/ 2*a* | 0.480(6) 0.4614(16) 0.656(3) | 0.49095 0.45565 0.65446 |
|  |  |  |  |  | C3/ 2*a* | 0.012(6) 0.2848(15) 0.442(4) | 0.01594 0.28358 0.43672 |
|  |  |  |  |  | C4/ 2*a* | 0.123(7) 0.2250(19) 0.002(4) | 0.11853 0.22925 0.00585 |
| Atom site occupancy | Fe1 | 0.92(2) | | 1 | 0.861(19) | | 1 |
| *Data collection* | | | | | | | |
| No. of measured, independent and observed [I > 2σ(I)] reflections | | 594/446/295 | |  | 934/860/762 | |  |
| R_int_ | | 2.22% | |  | 1.22% | |  |
| *Refinement* | | | | | | | |
| R_1_ | | 5.73% | |  | 2.98% | |  |
| wR_2_ | | 16.24% | |  | 7.08% | |  |
| GOF | | 0.945 | |  | 1.014 | |  |
| No. of reflections/No. of parameters | | 446/54 | |  | 860/103 | |  |


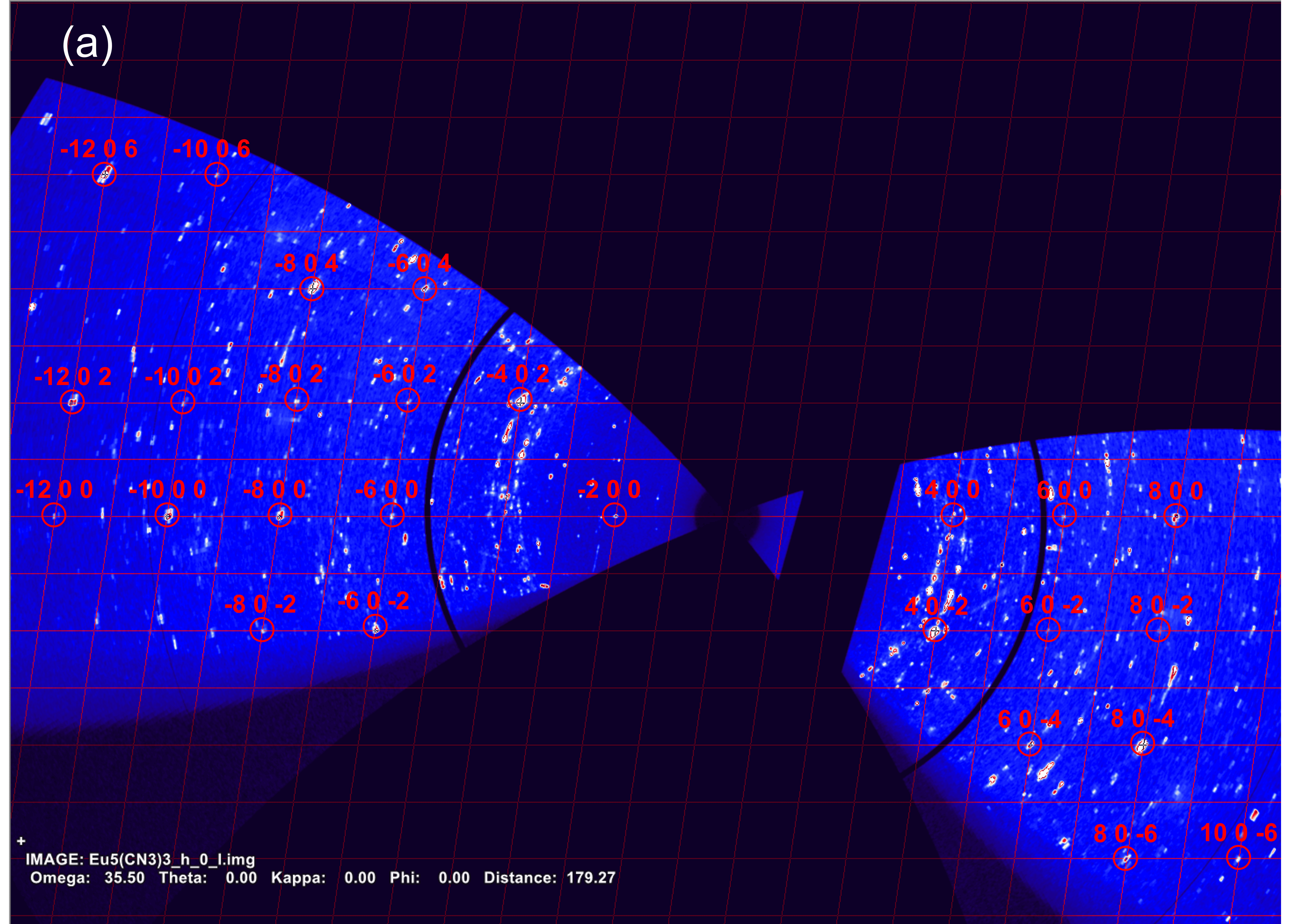


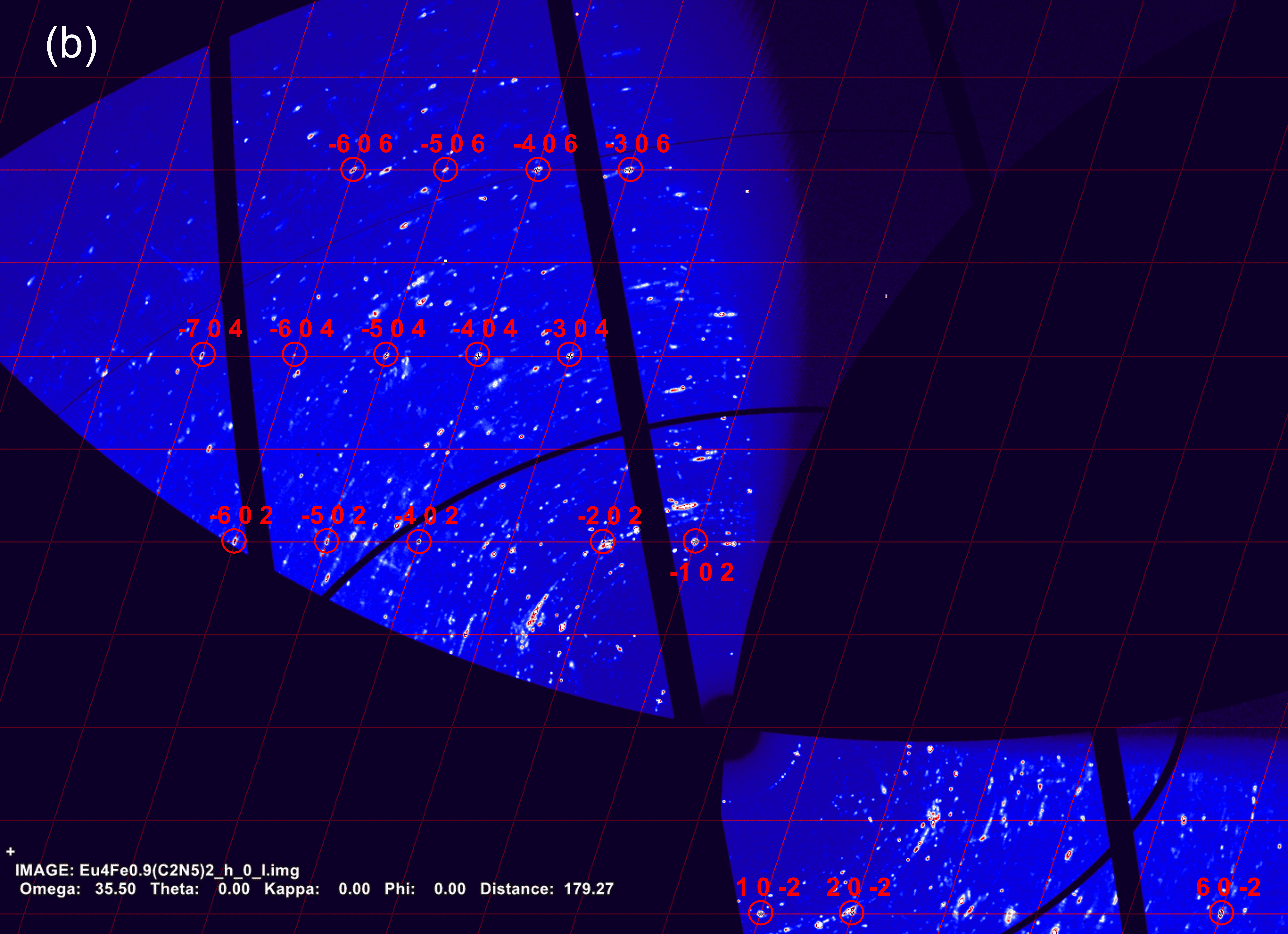


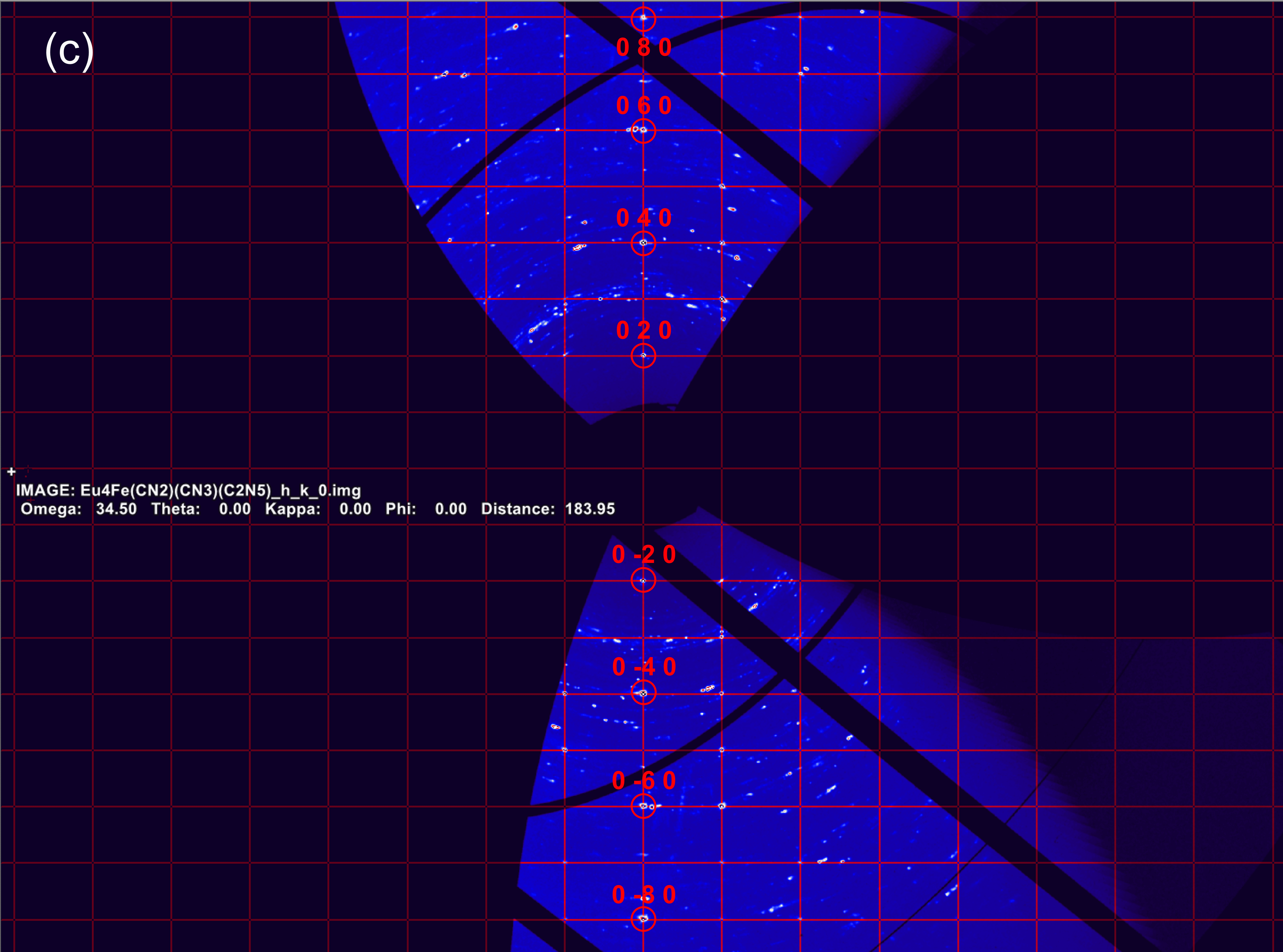


**Figure S1.** Reconstructed reciprocal lattice planes of the Eu–C–N and Eu–Fe–C–N compounds for the most representative domains: (a) Eu_5_(CN_3_)_3_, (b) Eu_4_Fe_0.909(16)_(C_2_N_5_)_2_, and (c) Eu_4_Fe_0.861(19)_(CN_2_)(CN_3_)(C_2_N_5_). Indexing was done using CrysAlis^Pro^ software.


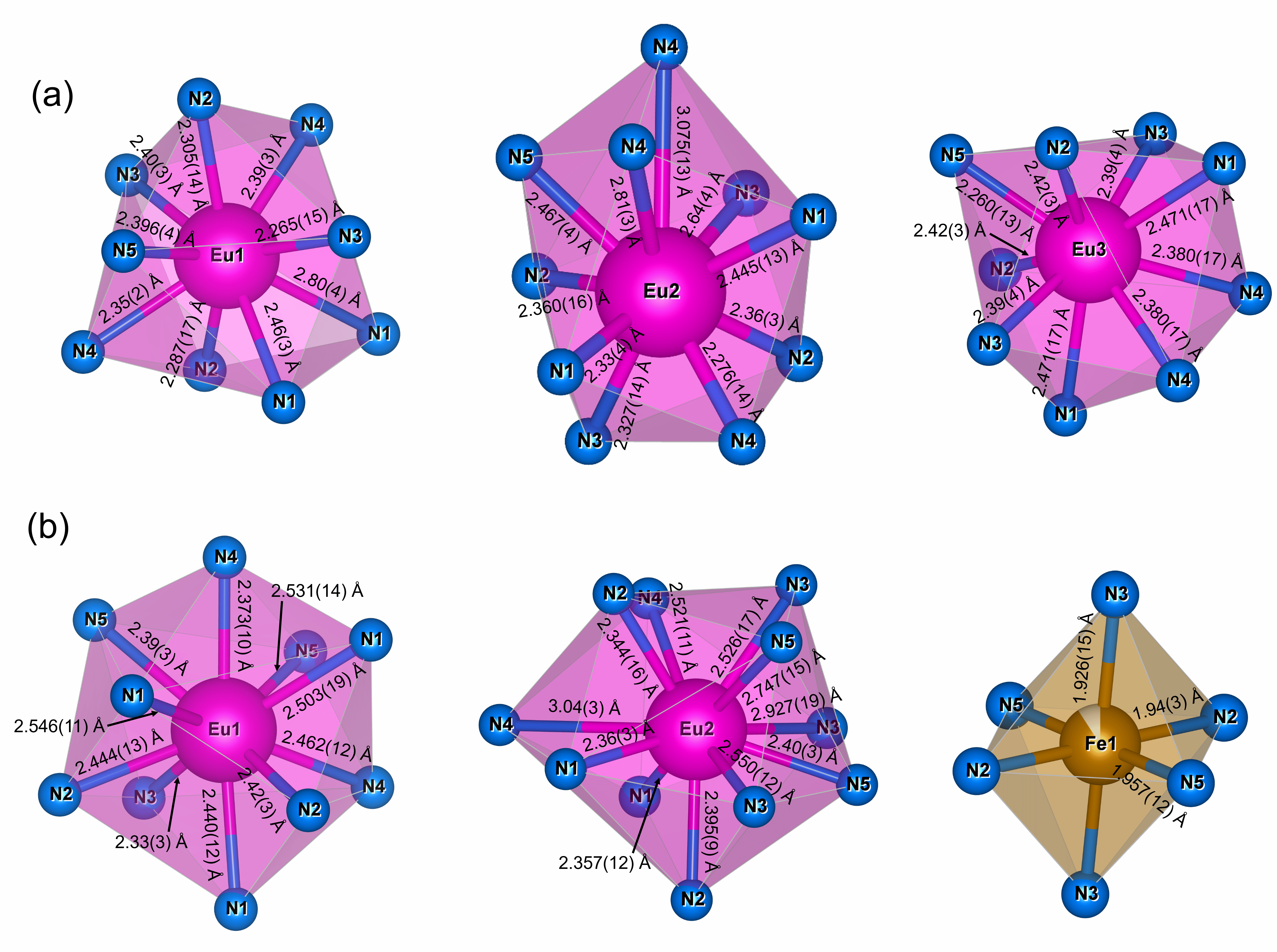


**Figure S2.** Coordination environment of metal atoms in (a) Eu_5_(CN_3_)_3_ and (b) Eu_4_Fe_0.909(16)_(C_2_N_5_)_2_ at a synthesis pressure of 50(3) GPa.


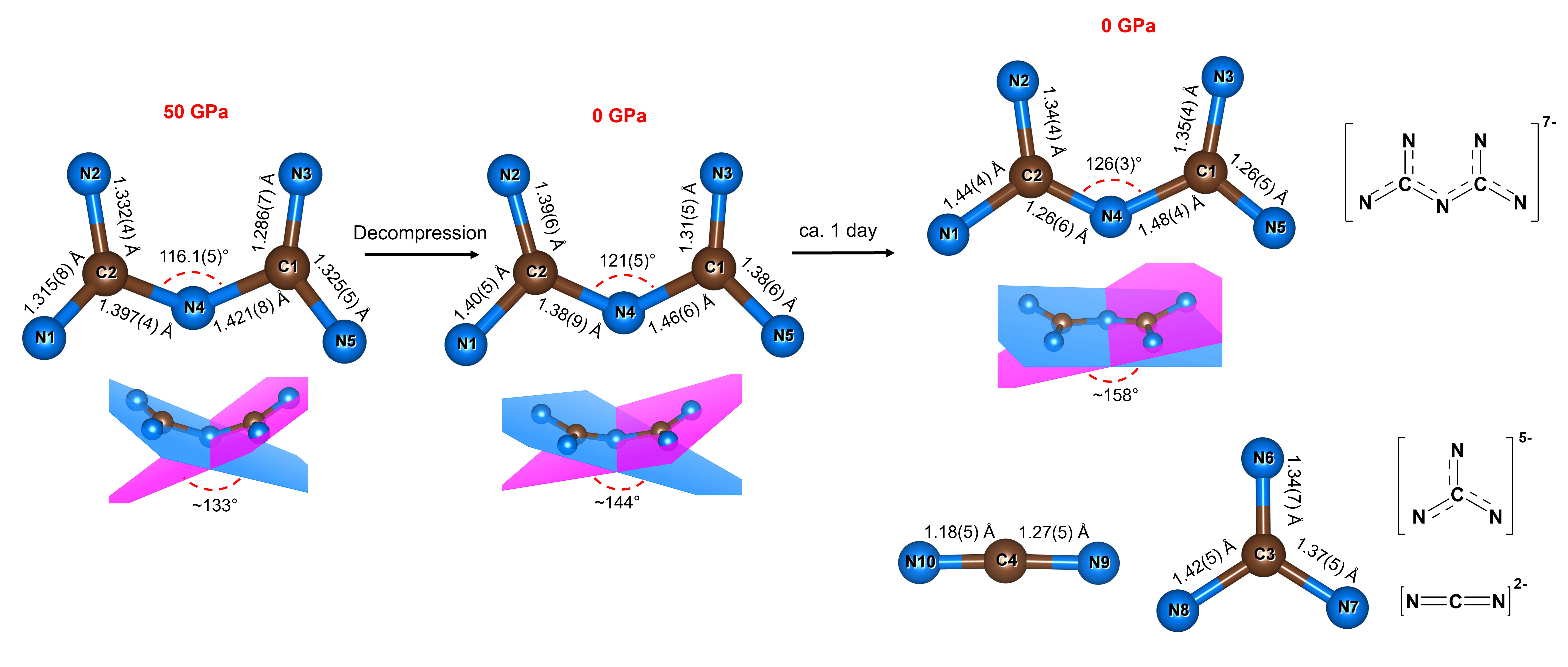


**Figure S3.** The pyronitridocarbonate anion [C_2_N_5_]^7-^ geometry during decompression of the DAC. At ambient conditions, there is a chemical transformation with half of the [C_2_N_5_]^7-^ anions splitting into guanidinate [CN_3_]^5-^ and carbodiimide [CN_2_]^2-^ anions.


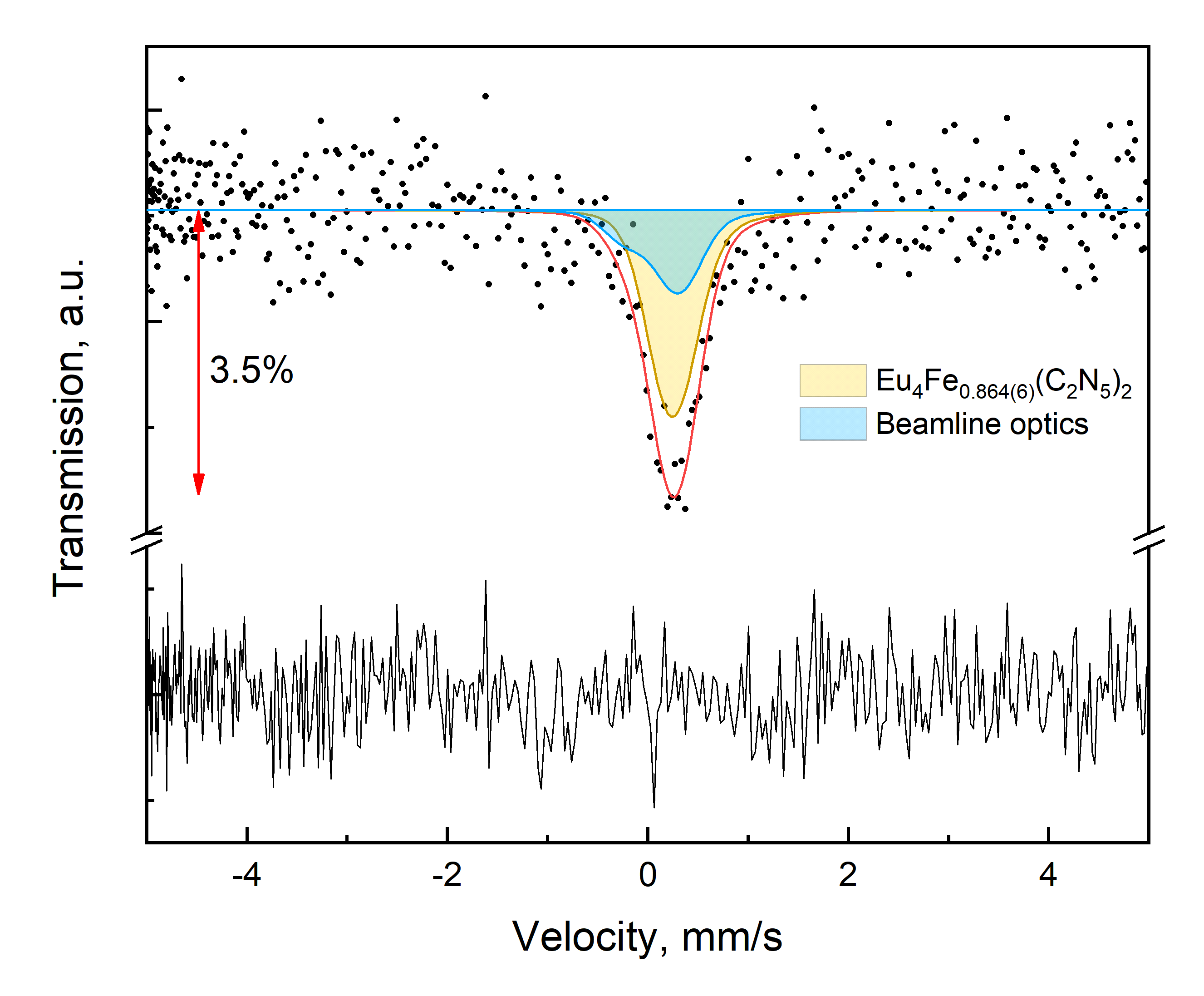


**Figure S4.** Synchrotron Mössbauer Source spectrum of Eu_4_Fe_0.864(6)_(C_2_N_5_)_2_ at 50(3) GPa. The iron contribution is fitted to a singlet (yellow) with a center shift of 0.26 mm/s. The red solid line represents the full transmission integral fit. The spectrum includes a contribution from iron impurities in the Be compound refractive lenses (blue) used to reduce the divergence of the incident beam on the Synchrotron Mössbauer Source.

**Supplementary DFT calculation results**

**Table S7.** VASP pseudopotentials used for different elements in the studied compounds, and the values of the effective Hubbard *U* parameter in DFT+*U* used for the magnetic elements.

| Compound | Functional | Element | Electron configuration | Number of valence electrons | *U_eff_* = *U*– *J* (eV) |
| --- | --- | --- | --- | --- | --- |
| Eu_4_Fe(C_2_N_5_)_2_ | GGA-PBE  PBEsol | Eu  Fe  C  N | [Kr-4d^10^]5s^2^5p^6^4f^7^6s^2^  [Ne-3s^2^]3p^6^3d^7^4s^1^  [He]2s^2^2p^2^  [He]2s^2^2p^3^ | 17  14  4  5 | 7  5  0  0 |
| Eu_4_Fe(CN_2_)(CN_3_)(C_2_N_5_) | GGA-PBE  PBEsol | Eu  Fe  C  N | [Kr-4d^10^]5s^2^5p^6^4f^7^6s^2^  [Ne-3s^2^]3p^6^3d^7^4s^1^  [He]2s^2^2p^2^  [He]2s^2^2p^3^ | 17  14  4  5 | 7  5  0  0 |
| Eu_5_(CN_3_)_3_ | GGA-PBE  PBEsol | Eu  C  N | [Kr-4d^10^]5s^2^5p^6^4f^7^6s^2^  [He]2s^2^2p^2^  [He]2s^2^2p^3^ | 17  4  5 | 7  0  0 |
| Eu_3_O_2_(CN_3_) | GGA-PBE  PBEsol | Eu  O  C  N | [Kr-4d^10^]5s^2^5p^6^4f^7^6s^2^  [He]2s^2^2p^4^  [He]2s^2^2p^2^  [He]2s^2^2p^3^ | 17  6  4  5 | 7  0  0  0 |

**Table S8.** DFT+*U* optimized crystallographic information for Eu_5_(CN_3_)_3_ compared with their corresponding experimental values at 50 GPa using both the PBE+*U* and PBEsol+*U* functionals. The errors in the calculated lattice parameters relative to the experimental values are given in parentheses.

| Eu_5_(CN_3_)_3_ | Experiment | PBE+*U* (Error %) | PBEsol+*U* (Error %) |
| --- | --- | --- | --- |
| Pressure (GPa) | 50(3) | 50 | |
| a (Å) | 10.318(14) | 10.3064  (0.11%) | 10.1906  (1.23%) |
| b (Å) | 6.3045(7) | 6.3790  (1.17%) | 6.3369  (0.51%) |
| c (Å) | 10.0150(16) | 10.0370  (0.22%) | 9.9617  (0.53%) |
| α (°) | 90 | 90  (0%) | 90  (0%) |
| β (°) | 98.72(4) | 98.75  (0.03%) | 98.79  (0.07%) |
| γ (°) | 90 | 90  (0%) | 90  (0%) |
| V (Å^3^) | 643.9(9) | 652.20  (1.27%) | 635.73  (1.28%) |
| Space group | *C*2/*c*, #15 | *C*2/*c*, #15 | *C*2/*c*, #15 |
| Wyckoff positions (magnetic atoms) and corresponding magnetic moments |  | Eu^1^(4f), 6 𝜇_𝐵_  Eu^2^(4f), 6 𝜇_𝐵_  Eu^3^(2e), 6 𝜇_𝐵_ | Eu^1^(4f), 6 𝜇_𝐵_  Eu^2^(4f), 6 𝜇_𝐵_  Eu^3^(2e), 6 𝜇_𝐵_ |
| Band Gap (eV) |  | 1.39 | 1.40 |

**Table S9.** DFT+*U* optimized crystallographic information for Eu_4_Fe(C_2_N_5_)_2_ and Eu_4_Fe(CN_2_)(CN_3_)(C_2_N_5_) compared with their corresponding experimental values at selected pressures of 50 GPa and 1 bar using both the PBE+*U* and PBEsol+*U* functionals. The errors in the calculated lattice parameters relative to the experimental values are given in parentheses.

|  | Eu_4_Fe(C_2_N_5_)_2_ | | | Eu_4_Fe(CN_2_)(CN_3_)(C_2_N_5_) | | |
| --- | --- | --- | --- | --- | --- | --- |
|  | Experiment | PBE+*U* (Error %) | PBEsol+*U* (Error %) | Experiment | PBE+*U* (Error %) | PBEsol+*U* (Error %) |
| Pressure | 50(3) GPa | 50 GPa | | 1 bar | | |
| a (Å) | 6.675(16) | 6.6718  (0.05%) | 6.6209  (0.81%) | 6.880(5) | 6.9009  (0.30%) | 6.8291  (0.74%) |
| b (Å) | 8.1521(14) | 8.2282  (0.92%) | 8.1567  (0.06%) | 9.0990(7) | 9.4373  (3.58%) | 9.2170  (1.28%) |
| c (Å) | 6.361(3) | 6.3262  (0.55%) | 6.2682  (1.46%) | 7.2599(19) | 7.3277  (0.93%) | 7.2201  (0.55%) |
| α (°) | 90 | 90  (0%) | 90  (0%) | 90 | 90  (0%) | 90  (0%) |
| β (°) | 108.26(12) | 107.81  (0.42%) | 107.93  (0.30%) | 108.14(5) | 107.28  (0.80%) | 108.18  (0.04%) |
| γ (°) | 90 | 90  (0%) | 90  (0%) | 90 | 90  (0%) | 90  (0%) |
| V (Å^3^) | 328.7(8) | 330.65  (0.59%) | 322.07  (2.02%) | 431.9(3) | 455.68  (5.22%) | 431.76  (0.03%) |
| Space group | *P*2_1_/*c*, #14 | | | *P*2_1_, #4 | | |
| Wyckoff positions (magnetic atoms) and corresponding magnetic moments |  | Eu^1^(4e), 6 𝜇_𝐵_  Eu^2^(4e), 6 𝜇_𝐵_  Fe(2a), 3.5 𝜇_𝐵_ | Eu^1^(4e), 6 𝜇_𝐵_  Eu^2^(4e), 6 𝜇_𝐵_  Fe(2a), 3.5 𝜇_𝐵_ |  | Eu^1^(2a), 6 𝜇_𝐵_  Eu^2^(2a), 6 𝜇_𝐵_  Eu^3^(2a), 7 𝜇_𝐵_  Eu^4^(2a), 6 𝜇_𝐵_  Fe(2a), 4 𝜇_𝐵_ | Eu^1^(2a), 6 𝜇_𝐵_  Eu^2^(2a), 6 𝜇_𝐵_  Eu^3^(2a), 7 𝜇_𝐵_  Eu^4^(2a), 6 𝜇_𝐵_  Fe(2a), 4 𝜇_𝐵_ |
| Band Gap (eV) |  | 0.45 | 0.41 |  | 0.39 | 0.31 |

**Table S10.** The magnetic properties calculated for Eu_4_Fe(C_2_N_5_)_2_ at 50 GPa: the spin orientations and the magnetic moments calculated for different magnetic configurations using a collinear method; the static enthalpy difference ΔH at 0 K with respect to the FM state; the magnetic moment on each sublattice of Eu, Fe.

| Eu_4_Fe(C_2_N_5_)_2_ (P = 50 GPa) | FM | AFM-I | AFM-II |
| --- | --- | --- | --- |
| Enthalpy, H (eV) | -237.2327 | -237.2141 | -236.8722 |
| P*V (eV) | 103.1945 | 103.1897 | 103.2077 |
| ΔH (0 K) (meV/mag. atom) | 0 | 1.87 | 36.06 |
| Wyckoff positions (magnetic atoms) and corresponding magnetic moments | Eu^1^(4e), +6 𝜇_𝐵_  Eu^2^(4e), +6 𝜇_𝐵_  Fe(2a), +3.5 𝜇_𝐵_ | Eu^1^(4e), +6 𝜇_𝐵_  Eu^2^(4e), -6 𝜇_𝐵_  Fe(2a), +3.5 𝜇_𝐵_ | Eu^1^(4e), +6 𝜇_𝐵_  Eu^2^(4e), -6 𝜇_𝐵_  Fe(2a), -3.5 𝜇_𝐵_ |


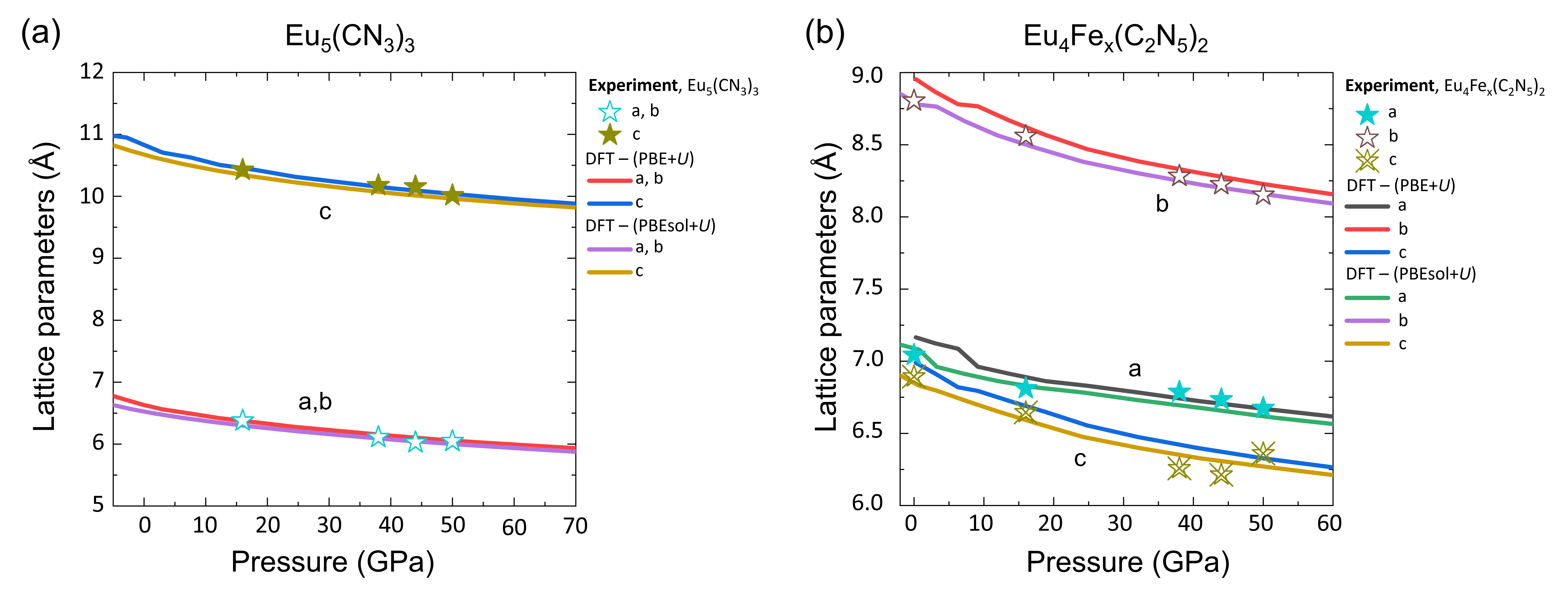


**Figure S5.** Experimental and calculated lattice parameters as a function of pressure for (a) Eu_5_(CN_3_)_3_ and (b) Eu_4_Fe_x_(C_2_N_5_)_2_. Experimental lattice constants were obtained from the SCXRD data collection and are presented by star symbols, whereas the DFT+*U* calculated ones are shown as solid lines, using both PBE+*U* and PBEsol+*U* functionals, respectively.


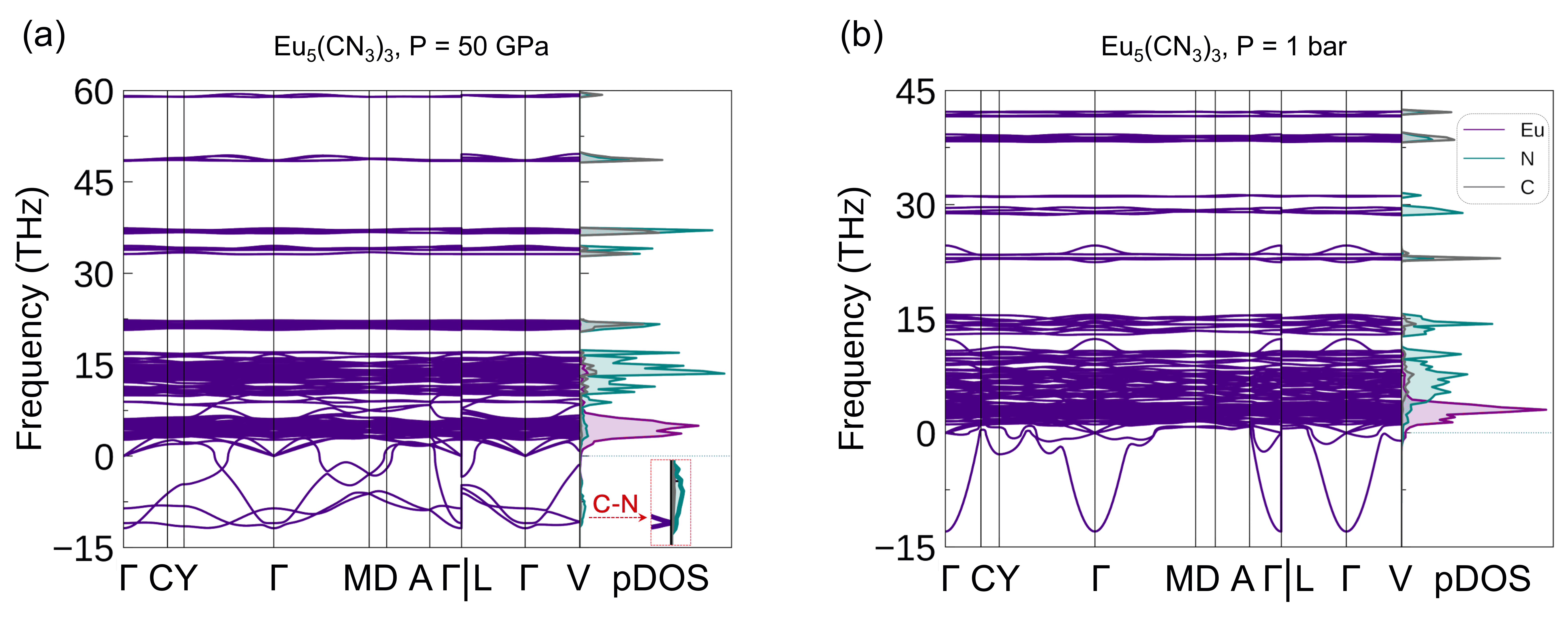


**Figure S6.** (a)-(b) The phonon dispersion relations for Eu_5_(CN_3_)_3_ at 50 GPa and 1 bar using harmonic approximation. Eu_5_(CN_3_)_3_ remains dynamically unstable both at 50 GPa and 1 bar. The instability in Eu_5_(CN_3_)_3_ is due to C–N bond asymmetry in the CN_3_ anions in a static lattice representation (T = 0 K). Eu_5_(CN_3_)_3_ is dynamically stable at room temperature (T = 300 K) as evident from our sTDEP calculations, presented in the main text of this paper.


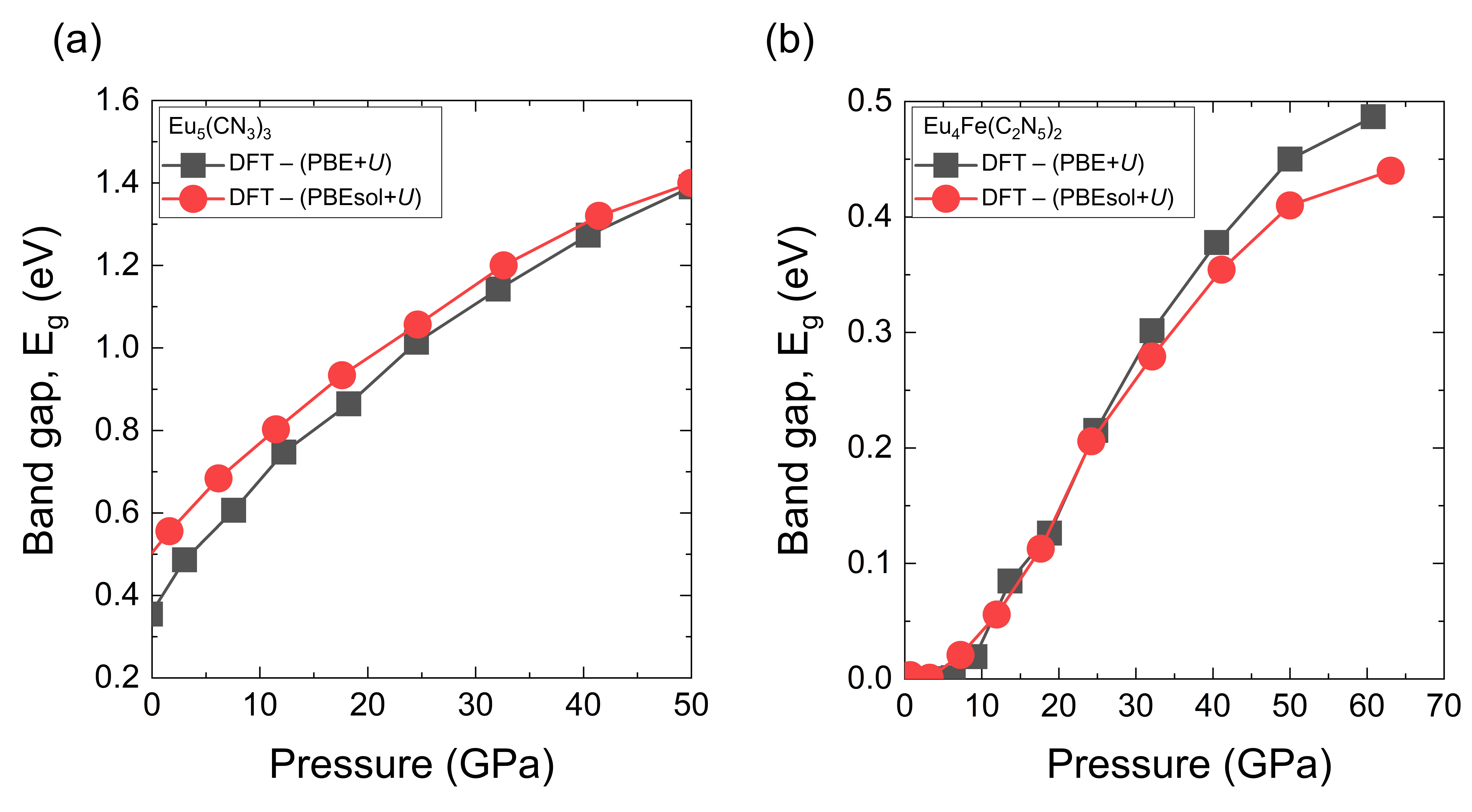


**Figure S7.** DFT+U calculated fundamental band gap, E_g_ in eV for (a) Eu_5_(CN_3_)_3_ and (b) Eu_4_Fe(C_2_N_5_)_2_ as a function of pressure. The variations in the fundamental energy gap were extracted both using the PBE+*U* and PBEsol+*U* methods as a function of pressure. An electronic transition from an insulating state to a metallic state occurs in the case of Eu_4_Fe(C_2_N_5_)_2_ below a pressure of 10 GPa.


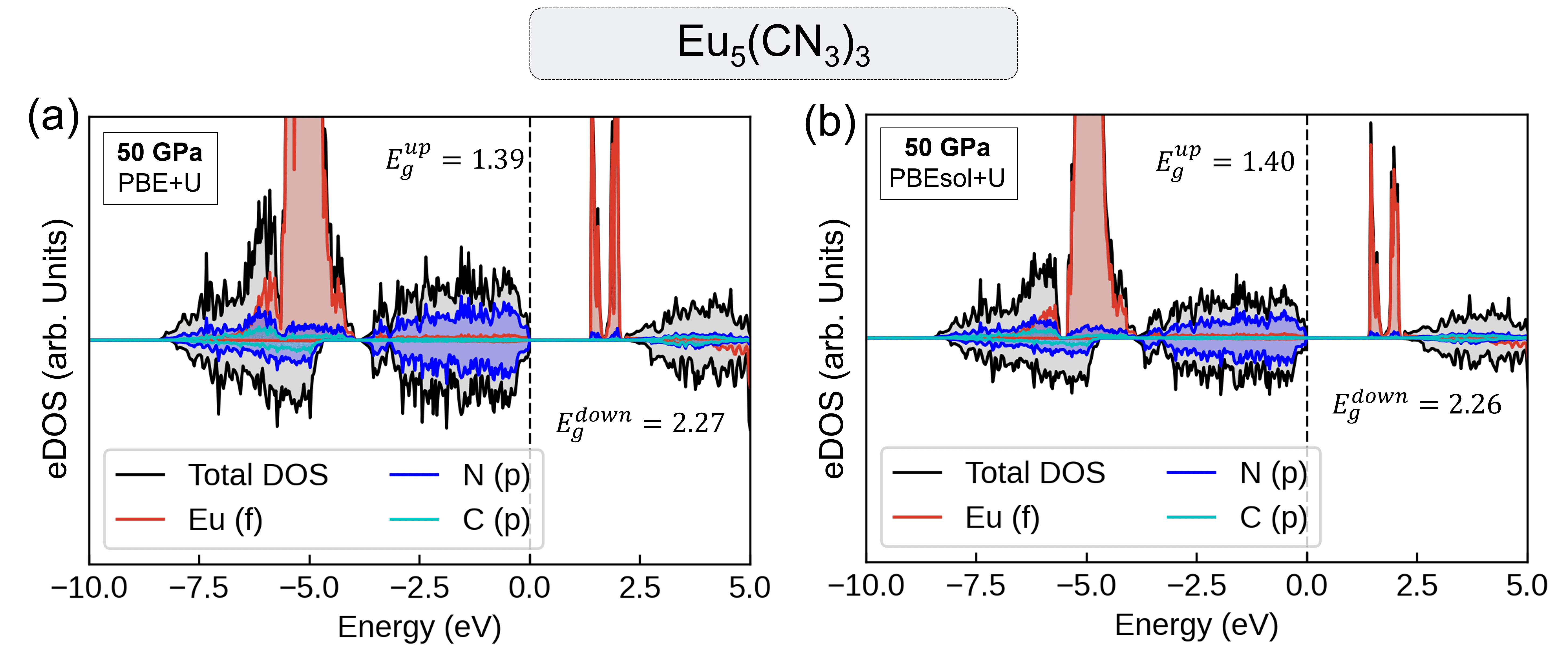


**Figure S8.** eDOS for Eu_5_(CN_3_)_3_ at 50 GPa using (a) PBE+U and (b) PBEsol+U functionals.


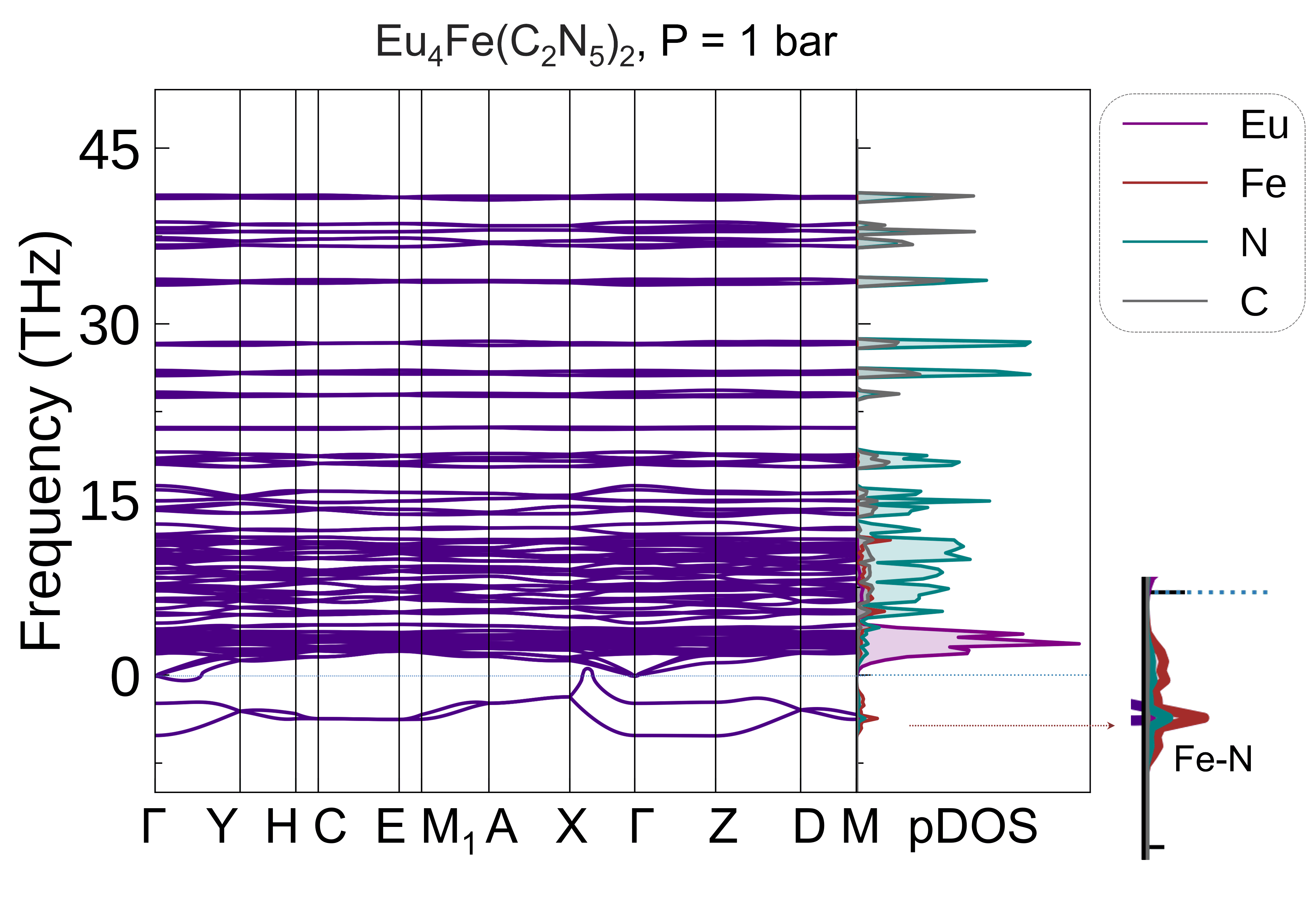


**Figure S9.** Calculated phonon dispersion relations and the phonon density of states (pDOS) for Eu_4_Fe(C_2_N_5_)_2_ at ambient pressure in space group (S.G.) symmetry of P2_1_/c (#14). The presence of two imaginary phonon branches is primarily attributed to the vibrations of Fe–N bonds, which exhibit weakened interactions at lower pressure. Consequently, the decomposition of Eu_4_Fe(C_2_N_5_)_2_ (P2_1_/c) at 1 bar results in the formation of a new phase, Eu_4_Fe(CN_2_)(CN_3_)(C_2_N_5_), characterized by the space group symmetry of P2_1_ (#4). This low symmetry Eu_4_Fe(CN_2_)(CN_3_)(C_2_N_5_) (P2_1_) phase is dynamically stable at ambient pressure, as discussed in the main text of this paper.


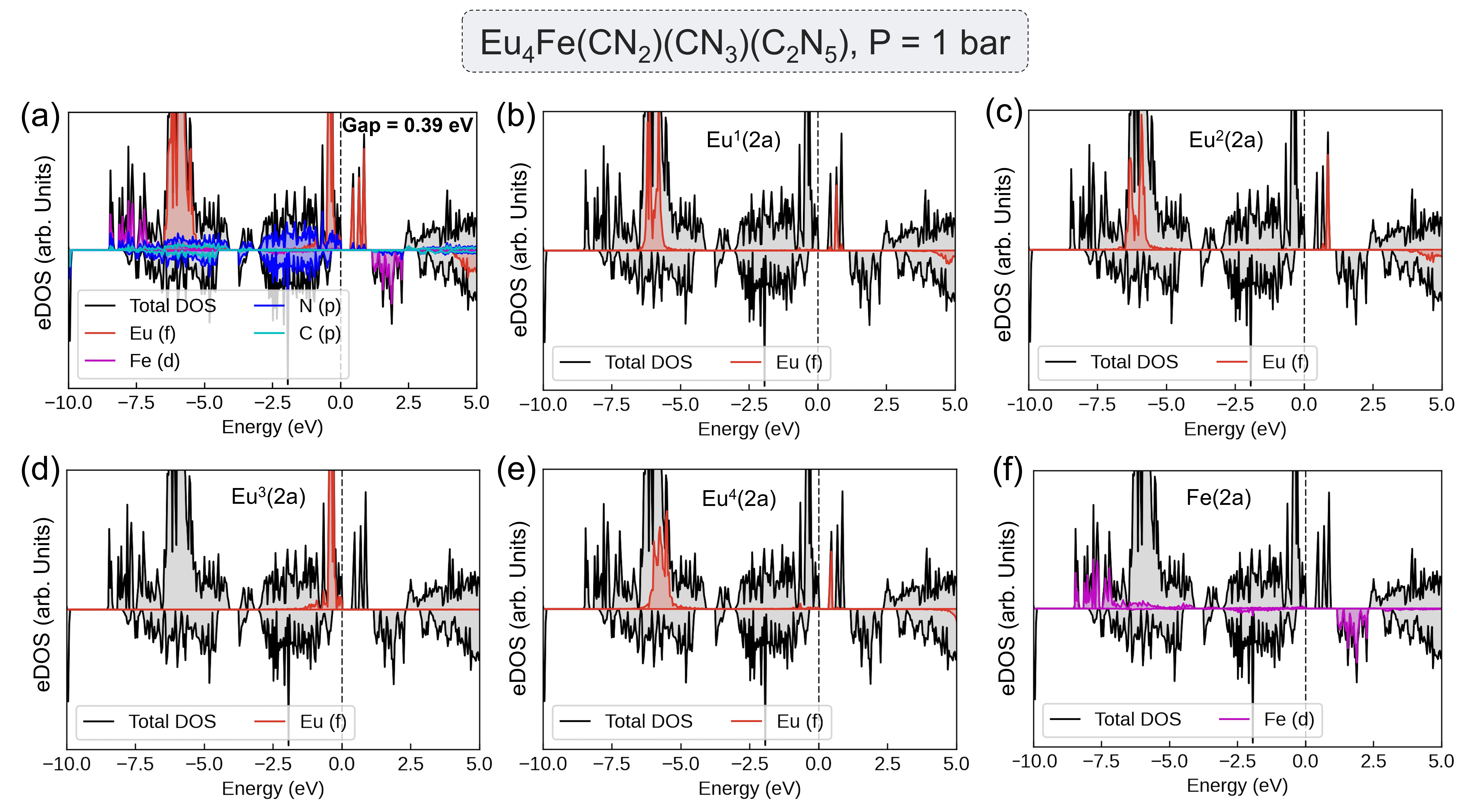


**Figure S10.** eDOS for Eu_4_Fe(CN_2_)(CN_3_)(C_2_N_5_) at 1 bar projected into four different Eu-Wyckoff sites (Eu^1^, Eu^2^, Eu^3^, Eu^4^) and one Fe (2a) site.

**Discussion S1:** PBEsol+*U* yields a similar eDOS but slightly smaller bandgaps (Figure S11a, b, Table S9). Upon decompression to ambient pressure, Eu_4_Fe(C_2_N_5_)_2_ undergoes a transition from an insulating to a half-metallic state, with Eu 4*f* states crossing the Fermi level in the spin-up channel while the spin-down channel remains gapped (1.23 eV) (Figure S11c). A similar trend in eDOS is observed at 1 bar using PBEsol+*U* (Figure S11d). In Eu_4_Fe(C_2_N_5_)_2_, the fundamental electronic gap between electron-hole states narrows monotonically with pressure release, closing near ~8 GPa using PBE+*U* and ~4 GPa using PBEsol+*U* before the system becomes half-metallic (Figure S7b).

It is important to note that though the position of Eu 4*f* states in the eDOS is highly sensitive to the Hubbard *U* parameter, the qualitative picture presented in the main text is not affected by the choice of *U*. Figure S12 illustrates the effect of a varying *U* on the eDOS of Eu_4_Fe(C_2_N_5_)_2_ using *U* = 5, 7, and 9 eV. A larger *U* pushes Eu 4*f* states far-off from the Fermi energy, while a smaller *U* brings them closer. Eu_4_Fe(C_2_N_5_)_2_ becomes metallic at *U* = 5 eV, whereas the bandgap increases significantly to 1.26 eV using *U* = 9 eV. The effect of *U* on the eDOS of Eu_5_(CN_3_)_3_ is similar to that observed in the case of Eu_4_Fe(C_2_N_5_)_2_, with a key distinction that Eu_5_(CN_3_)_3_ remains insulating even at a smaller *U* = 5 eV (Figure S13). For consistency in computational parameters, *U* = 7 eV was used for europium atoms in all studied compounds.

The optimized crystal structure of Eu_4_Fe(C_2_N_5_)_2_ at 50 GPa, in a collinear ferromagnetic (FM) configuration, was further used to generate two antiferromagnetic (AFM-I, AFM-II) configurations, wherein the spin orientation of the Eu1(4*e*) and Eu2(4*e*) Wyckoff positions were reversed relative to the Fe(2*a*) sublattice (Figure S14). A relative enthalpy comparison between the FM and AFM (I and II) states indicates that FM ordering is the energetically preferred magnetic ground state at 0 K (Table S10). Furthermore, the Mössbauer spectrum of Eu_4_Fe(C_2_N_5_)_2_ at 50 GPa exhibits no spin splitting, confirming that the compound remains paramagnetic at room temperature (Figure S4). Notably, Eu_4_Fe(C_2_N_5_)_2_ remains insulating in both FM and AFM configurations, with varying energy gaps for majority and minority spin states, as illustrated in the electronic density of states (Figure S14). Unless otherwise stated, all theoretical calculations in this study assume FM ordering for the magnetic atoms.

In Eu_4_Fe(C_2_N_5_)_2_, europium occupies two distinct Wyckoff positions, Eu1(4*e*) and Eu2(4*e*), hence giving rise to two distinct Eu 4*f* peaks, each contributing one electron to the unoccupied 4*f* states above the Fermi level (Figure S15b, c). Consequently, each europium site carries a magnetic moment of 6*μ_B_*. In Eu_4_Fe(C_2_N_5_)_2_, the Fe 3*d-*states are distributed over a broad energy range in the spin-up channel, extending from -10 to -1 eV. In contrast, the spin-down Fe 3*d*-states are very localized near the Fermi energy and strongly hybridized with N 2*p*-states, contributing significantly to the valence band edge (Figure S15d). Each iron site in Eu_4_Fe(C_2_N_5_)_2_ possesses a magnetic moment of 3.5*μ_B_* at the Fe(2*a*) sublattice.


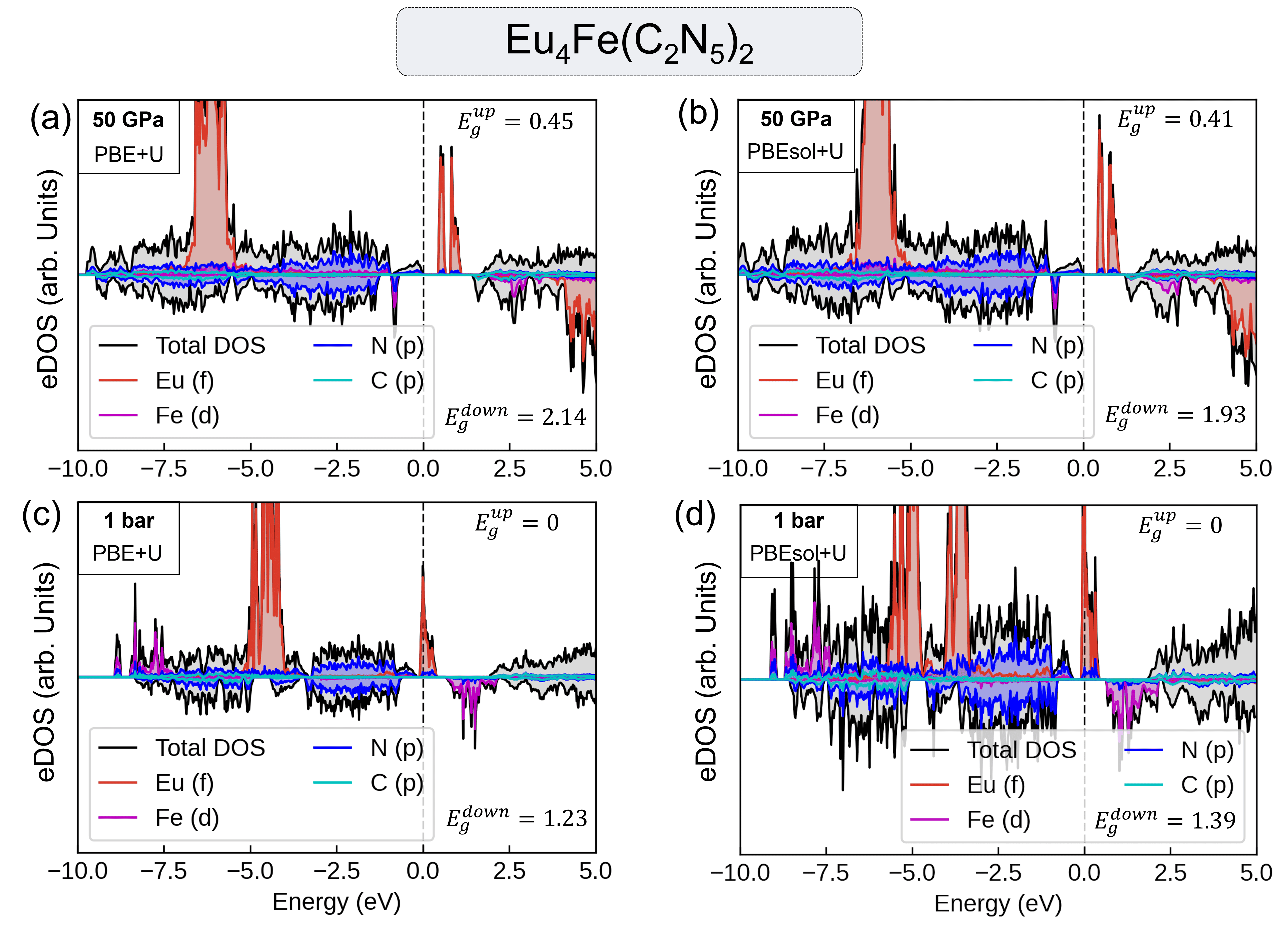


**Figure S11.** eDOS for Eu_4_Fe(C_2_N_5_)_2_ at 50 GPa and 1 bar using PBE+U and PBEsol+U functionals.


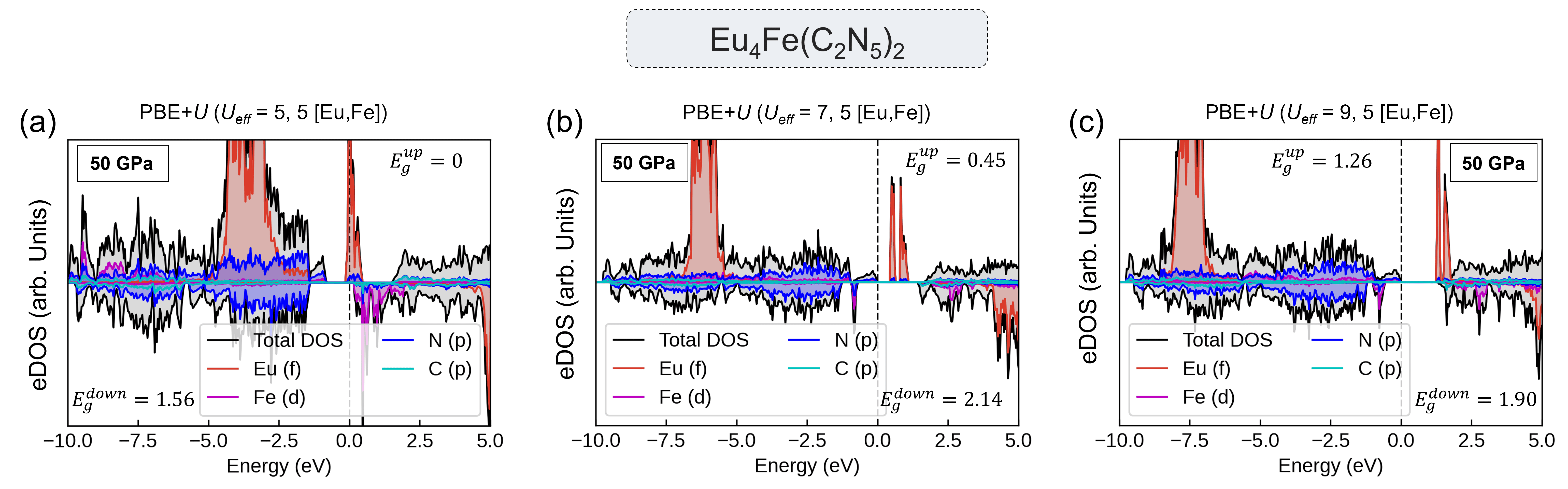


**Figure S12.** The effect of Hubbard U parameter in PBE+U calculations on the electronic structure of Eu_4_Fe(C_2_N_5_)_2_ at 50 GPa.


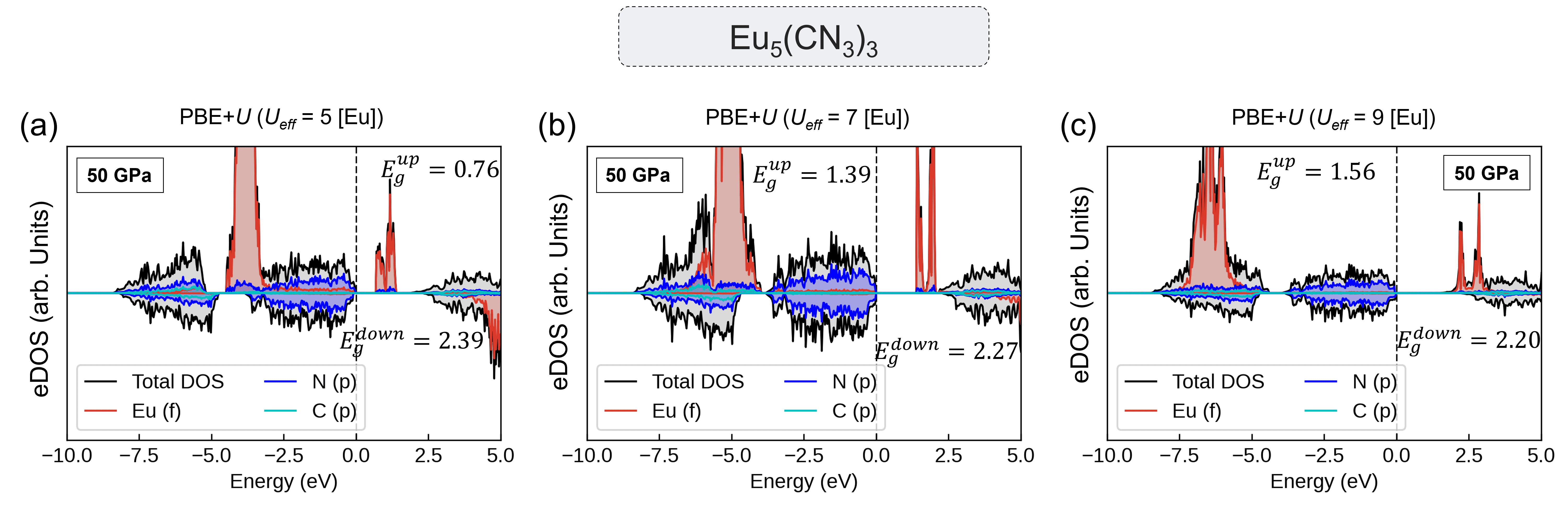


**Figure S13.** The effect of Hubbard U parameter in PBE+U calculations on the electronic eDOS of Eu_5_(CN_3_)_3_ at 50 GPa.


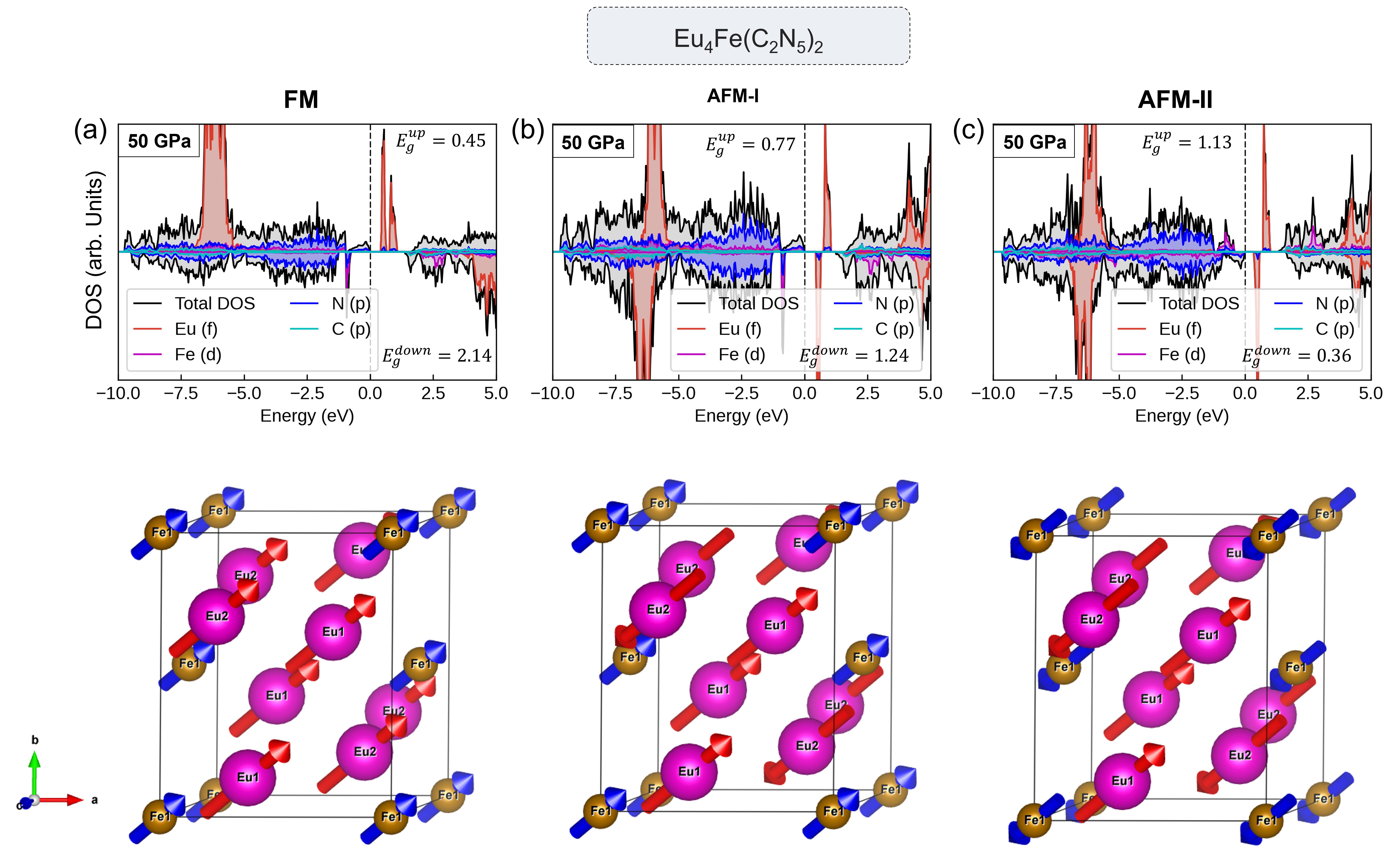


**Figure S14.** eDOS for Eu_4_Fe(C_2_N_5_)_2_ at 50 GPa in different magnetic configurations for Eu and Fe, such as FM, AFM-I, and AFM-II using PBE+*U* functionals. The static enthalpy difference at 0 K and the magnetic moment on each sublattice of Eu, Fe are summarized in Table S10.


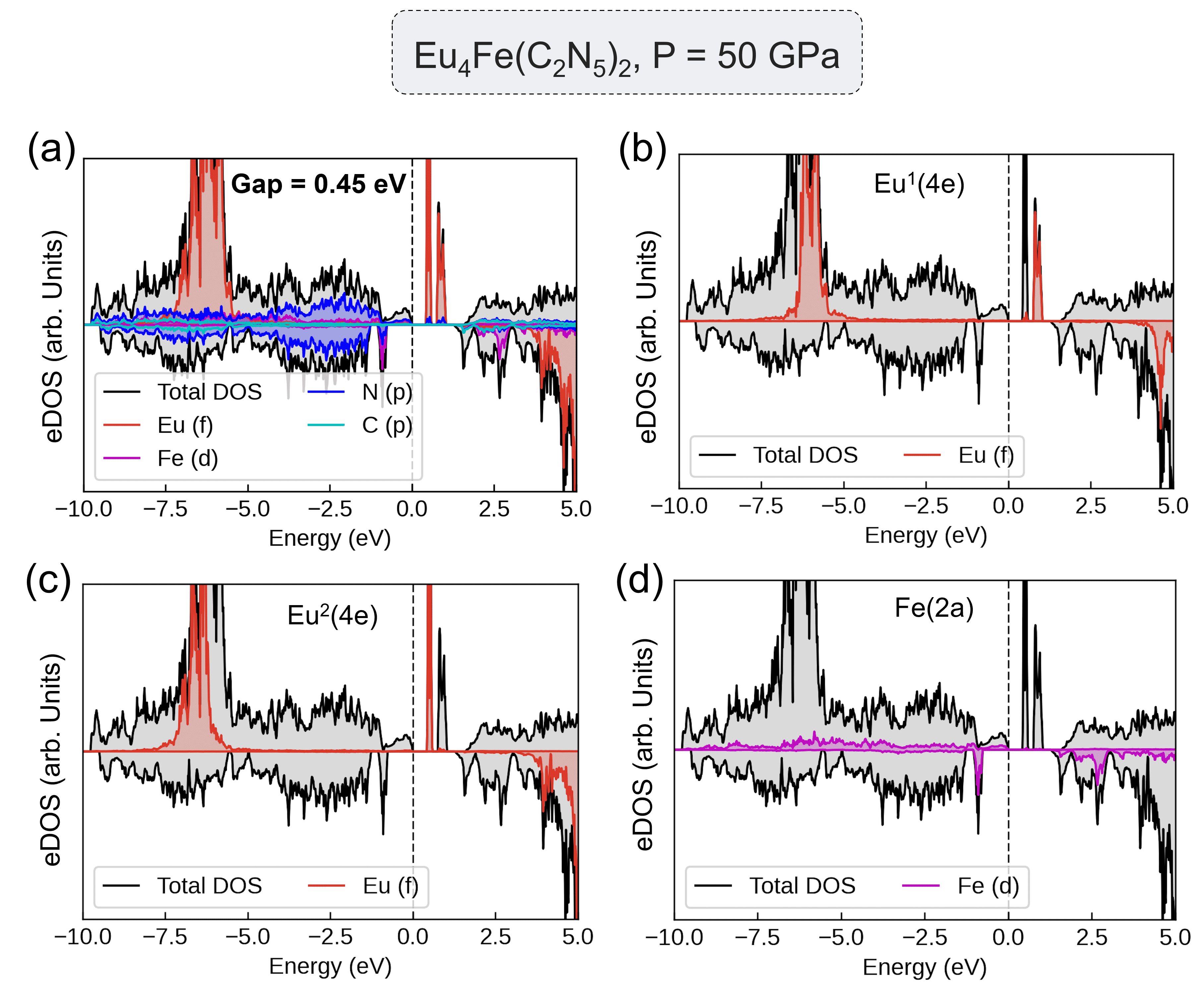


**Figure S15.** eDOS for Eu_4_Fe(C_2_N_5_)_2_ at 50 GPa projected into two different Eu-Wyckoff sites (Eu^1^, Eu^2^) and one Fe (2a) site.

**References**

(1) Kantor, I.; Prakapenka, V.; Kantor, A.; Dera, P.; Kurnosov, A.; Sinogeikin, S.; Dubrovinskaia, N.; Dubrovinsky, L. BX90: A New Diamond Anvil Cell Design for X-Ray Diffraction and Optical Measurements. *Rev. Sci. Instrum.* **2012**, *83* (12), 125102. https://doi.org/10.1063/1.4768541.

(2) Flosbach, N. T. Systematic Studies of Oxoanionic Lanthanoid Salts in Their + II and + IV Oxidation States. 2024, p 230.

(3) Wandner, D.; Link, P.; Heyer, O.; Mydosh, J.; Ahmida, M. A.; Abd-Elmeguid, M. M.; Speldrich, M.; Lueken, H.; Ruschewitz, U. Structural Phase Transitions in EuC_2_. *Inorg. Chem.* **2010**, *49* (1), 312–318. https://doi.org/10.1021/ic901979v.

(4) Aprilis, G.; Strohm, C.; Kupenko, I.; Linhardt, S.; Laskin, A.; Vasiukov, D. M.; Cerantola, V.; Koemets, E. G.; McCammon, C.; Kurnosov, A.; Chumakov, A. I.; Rüffer, R.; Dubrovinskaia, N.; Dubrovinsky, L. Portable Double-Sided Pulsed Laser Heating System for Time-Resolved Geoscience and Materials Science Applications. *Rev. Sci. Instrum.* **2017**, *88* (8). https://doi.org/10.1063/1.4998985.

(5) Fedotenko, T.; Dubrovinsky, L.; Aprilis, G.; Koemets, E.; Snigirev, A.; Snigireva, I.; Barannikov, A.; Ershov, P.; Cova, F.; Hanfland, M.; Dubrovinskaia, N. Laser Heating Setup for Diamond Anvil Cells for in Situ Synchrotron and in House High and Ultra-High Pressure Studies. *Rev. Sci. Instrum.* **2019**, *90* (10), 104501. https://doi.org/10.1063/1.5117786.

(6) Akahama, Y.; Kawamura, H. Pressure Calibration of Diamond Anvil Raman Gauge to 310 GPa. *J. Appl. Phys.* **2006**, *100* (4), 043516. https://doi.org/10.1063/1.2335683.

(7) Anzellini, S.; Dewaele, A.; Occelli, F.; Loubeyre, P.; Mezouar, M. Equation of State of Rhenium and Application for Ultra High Pressure Calibration. *J. Appl. Phys.* **2014**, *115* (4), 043511. https://doi.org/10.1063/1.4863300.

(8) Akbar, F. I.; Aslandukov, A.; Yin, Y. High-Pressure Chemistry of H-N-F Ternary System [Dataset]. European Synchrotron Radiation Facility 2027. https://doi.org/10.15151/ESRF-ES-1685311408.

(9) Akbar, F. I.; Bykov, M.; Bykova, E.; Gontcharov, A. F.; Kovalev, V.; Sharapova, N.; Spahr, D. Synthetic Vacancy-Rich Iron Silicates as a Key for Understanding Shock Rock-Forming Processes [Dataset]. European Synchrotron Radiation Facility 2027. https://doi.org/10.15151/ESRF-ES-1901553724.

(10) Boehler, R. New Diamond Cell for Single-Crystal x-Ray Diffraction. *Rev. Sci. Instrum.* **2006**, *77* (11). https://doi.org/10.1063/1.2372734.

(11) Aslandukov, A.; Aslandukov, M.; Dubrovinskaia, N.; Dubrovinsky, L. *Domain Auto Finder* (*DAFi*) Program: The Analysis of Single-Crystal X-Ray Diffraction Data from Polycrystalline Samples. *J. Appl. Cryst.* **2022**, *55*, 1383–1391. https://doi.org/10.1107/s1600576722008081.

(12) Dolomanov, O. V.; Bourhis, L. J.; Gildea, R. J.; Howard, J. A. K.; Puschmann, H. *OLEX2*: A Complete Structure Solution, Refinement and Analysis Program. *J. Appl. Cryst.* **2009**, *42* (2), 339–341. https://doi.org/10.1107/S0021889808042726.

(13) Sheldrick, G. M. *SHELXT* – Integrated Space-Group and Crystal-Structure Determination. *Acta Cryst.* **2015**, *A71* (1), 3–8. https://doi.org/10.1107/S2053273314026370.

(14) Sheldrick, G. M. Crystal Structure Refinement with *SHELXL*. *Acta Cryst.* **2015**, *C71* (1), 3–8. https://doi.org/10.1107/S2053229614024218.

(15) Momma, K.; Izumi, F. *VESTA 3* for Three-Dimensional Visualization of Crystal, Volumetric and Morphology Data. *J. Appl. Cryst.* **2011**, *44* (6), 1272–1276. https://doi.org/10.1107/S0021889811038970.

(16) Gonzalez-Platas, J.; Alvaro, M.; Nestola, F.; Angel, R. *EosFit7-GUI*: A New Graphical User Interface for Equation of State Calculations, Analyses and Teaching. *J. Appl. Cryst.* **2016**, *49* (4), 1377–1382. https://doi.org/10.1107/S1600576716008050.

(17) Link, L.; Niewa, R. Polynator: A Tool to Identify and Quantitatively Evaluate Polyhedra and Other Shapes in Crystal Structures. *J. Appl. Cryst.* **2023**, *56* (6), 1855–1864. https://doi.org/10.1107/S1600576723008476.

(18) *Cambridge Crystallographic Data Centre and Fachinformationszentrum Karlsruhe Access Structures service*. www.ccdc.cam.ac.uk/structures.

(19) Rüffer, R.; Chumakov, A. I. Nuclear Resonance Beamline at ESRF. *Hyperfine Interact.* **1996**, *97*–*98* (1), 589–604. https://doi.org/10.1007/BF02150199.

(20) Potapkin, V.; Chumakov, A. I.; Smirnov, G. V.; Celse, J.-P.; Rüffer, R.; McCammon, C.; Dubrovinsky, L. The ^57^Fe Synchrotron Mössbauer Source at the ESRF. *J. Synchrotron Rad.* **2012**, *19* (4), 559–569. https://doi.org/10.1107/S0909049512015579.

(21) Yaroslavtsev, S. SYNCmoss Software Package for Fitting Mössbauer Spectra Measured with a Synchrotron Mössbauer Source. *J. Synchrotron Rad.* **2023**, *30* (3), 596–604. https://doi.org/10.1107/S1600577523001686.

(22) Kresse, G.; Furthmüller, J. Efficient Iterative Schemes for Ab Initio Total-Energy Calculations Using a Plane-Wave Basis Set. *Phys. Rev. B* **1996**, *54* (16), 11169. https://doi.org/10.1103/PhysRevB.54.11169.

(23) Blöchl, P. E. Projector Augmented-Wave Method. *Phys. Rev. B* **1994**, *50*, 17953–17979. https://doi.org/10.1103/PhysRevB.50.17953.

(24) Kresse, G.; Joubert, D. From Ultrasoft Pseudopotentials to the Projector Augmented-Wave Method. *Phys. Rev. B* **1999**, *59* (3), 1758–1775. https://doi.org/10.1103/PhysRevB.59.1758.

(25) Perdew, J. P.; Burke, K.; Ernzerhof, M. Generalized Gradient Approximation Made Simple. *Phys. Rev. Lett.* **1996**, *77* (18), 3865–3868. https://doi.org/10.1103/PhysRevLett.77.3865.

(26) Perdew, J. P.; Ruzsinszky, A.; Csonka, G. I.; Vydrov, O. A.; Scuseria, G. E.; Constantin, L. A.; Zhou, X.; Burke, K. Restoring the Density-Gradient Expansion for Exchange in Solids and Surfaces. *Phys. Rev. Lett.* **2008**, *100* (13), 136406. https://doi.org/10.1103/PhysRevLett.100.136406.

(27) Perdew, J. P.; Ruzsinszky, A.; Csonka, G. I.; Vydrov, O. A.; Scuseria, G. E.; Constantin, L. A.; Zhou, X.; Burke, K. Erratum: Restoring the Density-Gradient Expansion for Exchange in Solids and Surfaces [Phys. Rev. Lett. 100 , 136406 (2008)]. *Phys. Rev. Lett.* **2009**, *102* (3), 039902. https://doi.org/10.1103/PhysRevLett.102.039902.

(28) Monkhorst, H. J.; Pack, J. D. Special Points for Brillouin-Zone Integrations. *Phys. Rev. B* **1976**, *13* (12), 5188–5192. https://doi.org/10.1103/PhysRevB.13.5188.

(29) Blöchl, P. E.; Jepsen, O.; Andersen, O. K. Improved Tetrahedron Method for Brillouin-Zone Integrations. *Phys. Rev. B* **1994**, *49* (23), 16223–16233. https://doi.org/10.1103/PhysRevB.49.16223.

(30) Dudarev, S. L.; Botton, G. A.; Savrasov, S. Y.; Humphreys, C. J.; Sutton, A. P. Electron-Energy-Loss Spectra and the Structural Stability of Nickel Oxide: An LSDA+U Study. *Phys. Rev. B* **1998**, *57* (3), 1505–1509. https://doi.org/10.1103/PhysRevB.57.1505.

(31) Liu, B.-L.; Wang, Y.-C.; Liu, Y.; Xu, Y.-J.; Chen, X.; Song, H.-Z.; Bi, Y.; Liu, H.-F.; Song, H.-F. Comparative Study of First-Principles Approaches for Effective Coulomb Interaction Strength *U_Eff_* between Localized f-Electrons: Lanthanide Metals as an Example. *J. Chem. Phys.* **2023**, *158* (8). https://doi.org/10.1063/5.0137264.

(32) Djabri, K.; Bouafia, H.; Sahli, B.; Djebour, B.; Hiadsi, S.; Dorbane, A.; Abidri, B.; Latigui, A. Elastic Anisotropy, Electronic and Magnetic Behaviours of Ferromagnetic Europium Niobate EuNbO_3_ in Orthorhombic Structure: DFT + U, MFA and QTAIM Studies. *Philos. Mag.* **2020**, *100* (22), 2889–2911. https://doi.org/10.1080/14786435.2020.1798536.

(33) Meng, Y.; Liu, X.-W.; Huo, C.-F.; Guo, W.-P.; Cao, D.-B.; Peng, Q.; Dearden, A.; Gonze, X.; Yang, Y.; Wang, J.; Jiao, H.; Li, Y.; Wen, X.-D. When Density Functional Approximations Meet Iron Oxides. *J. Chem. Theory Comput.* **2016**, *12* (10), 5132–5144. https://doi.org/10.1021/acs.jctc.6b00640.

(34) Togo, A.; Chaput, L.; Tadano, T.; Tanaka, I. Implementation Strategies in Phonopy and Phono3py. *J. Phys.: Condens. Matter* **2023**, *35* (35), 353001. https://doi.org/10.1088/1361-648X/acd831.

(35) Togo, A. First-Principles Phonon Calculations with Phonopy and Phono3py. *J. Phys. Soc. Jpn.* **2023**, *92* (1), 012001. https://doi.org/10.7566/JPSJ.92.012001.

(36) Hellman, O.; Abrikosov, I. A.; Simak, S. I. Lattice Dynamics of Anharmonic Solids from First Principles. *Phys. Rev. B* **2011**, *84* (18), 180301. https://doi.org/10.1103/PhysRevB.84.180301.

(37) Hellman, O.; Steneteg, P.; Abrikosov, I. A.; Simak, S. I. Temperature Dependent Effective Potential Method for Accurate Free Energy Calculations of Solids. *Phys. Rev. B* **2013**, *87* (10), 104111. https://doi.org/10.1103/PhysRevB.87.104111.

(38) Hellman, O.; Abrikosov, I. A. Temperature-Dependent Effective Third-Order Interatomic Force Constants from First Principles. *Phys. Rev. B* **2013**, *88* (14), 144301. https://doi.org/10.1103/PhysRevB.88.144301.
